# Circulating cell-free and extracellular vesicles-derived microRNA as prognostic biomarkers in patients with early-stage NSCLC: results from RESTING study

**DOI:** 10.1186/s13046-024-03156-y

**Published:** 2024-08-22

**Authors:** Elisabetta Petracci, Luigi Pasini, Milena Urbini, Enriqueta Felip, Franco Stella, Fabio Davoli, Maurizio Salvi, Michele Beau-Faller, Michela Tebaldi, Irene Azzali, Matteo Canale, Piergiorgio Solli, Giulia Lai, Ramon Amat, Caterina Carbonell, Pierre-Emmanuel Falcoz, Alex Martinez-Marti, Erwan Pencreach, Angelo Delmonte, Lucio Crinò, Paola Ulivi

**Affiliations:** 1 Unit of Biostatistics and Clinical Trials, IRCCS Istituto Romagnolo per lo Studio dei Tumori (IRST) “Dino Amadori”, Meldola, Italy; 2 Biosciences Laboratory, IRCCS Istituto Romagnolo per lo Studio dei Tumori (IRST) “Dino Amadori”, Meldola, Italy; 3 Vall d’Hebron Institute of Oncology (VHIO), Barcelona, Spain; 4 Thoracic Surgery Department AUSL Romagna, Forlì, Italy; 5 Thoracic Surgery Department AUSL Romagna, Ravenna, Italy; 6 Thoracic Surgery Department AUSL Romagna, Riccione, Italy; 7 Molecular Laboratory, University Hospital, Strasbourg University, Strasburg, France; 8 Unit of Thoracic Surgery and Lung Transplantation, IRCCS Azienda Ospedaliero-Universitaria di Bologna, Bologna, Italy; 9 Thoracic Surgery Department, Nouvel Hôpital Civil’, University Hospital, Strasburg, France; 10 Oncology Department, Istituto Romagnolo per lo Studio dei Tumori “Dino Amadori” (IRST) IRCCS, Meldola, Italy

## Abstract

**Background:**

Factors to accurately stratify patients with early-stage non-small cell lung cancer (NSCLC) in different prognostic groups are still needed. This study aims to investigate 1) the prognostic potential of circulating cell-free (CF) and extracellular vesicles (EVs)-derived microRNA (miRNAs), and 2) their added value with respect to known prognostic factors (PFs).

**Methods:**

The RESTING study is a multicentre prospective observational cohort study on resected stage IA-IIIA patients with NSCLC. The primary end-point was disease-free survival (DFS), and the main analyses were carried out separately for CF- and EV-miRNAs. CF- and EV-miRNAs were isolated from plasma, and miRNA-specific libraries were prepared and sequenced. To reach the study aims, three statistical models were specified: one using the miRNA data only (Model 1); one using both miRNAs and known PFs (age, gender, and pathological stage) (Model 2), and one using the PFs alone (Model 3). Five-fold cross-validation (CV) was used to assess the predictive performance of each. Standard Cox regression and elastic net regularized Cox regression were used.

**Results:**

A total of 222 patients were enrolled. The median follow-up time was 26.3 (95% CI 25.4–27.6) months. From Model 1, three CF-miRNAs and 21 EV-miRNAs were associated with DFS. In Model 2, two CF-miRNAs (miR-29c-3p and miR-877-3p) and five EV-miRNAs (miR-181a-2-3p, miR-182-5p, miR-192-5p, miR-532-3p and miR-589-5p) remained associated with DFS. From pathway enrichment analysis, TGF-beta and NOTCH were the most involved pathways.

**Conclusion:**

This study identified promising prognostic CF- and EV-miRNAs that could be used as a non-invasive, cost-effective tool to aid clinical decision-making. However, further evaluation of the obtained miRNAs in an external cohort of patients is warranted.

**Supplementary Information:**

The online version contains supplementary material available at 10.1186/s13046-024-03156-y.

## Introduction

Lung cancer is one of the most common cancers and exhibits the highest mortality worldwide [1]. Significant progress has been made concerning metastatic non-small cell lung cancer (NSCLC) thanks to the introduction of targeted therapy and immunotherapy in clinical practice. Of note, these therapies have been incorporated in earlier stages.

Early-stage non-small cell lung cancer (ES-NSCLC) represents only 20–30% of all NSCLC and is characterized by a high survival probability after surgery. However, considering stage IA-IIIA NSCLC, an overall relapse rate of about 50% is observed with wide variations within the same tumor node metastasis (TNM) stage. Most recurrences happen within the first two years after the primary tumor resection, usually at distant sites, and are associated with a dismal 5-year survival of 30% [2]. Efforts are being made to identify molecular tumor characteristics and tumor microenvironment (TME) features that discriminate patients at higher risk of relapse [3–5]. Recurrent stage I lung adenocarcinomas exhibited a higher mutation load and a lower methylation profile with respect to non-recurrent tumors, as well as widespread activation of known cancer and cell cycle pathways. Moreover, recurrent tumors displayed downregulation of immune response pathways, including antigen presentation and Th1/Th2 activation [6]. With regard to circulating biomarkers, circulating tumor DNA levels evaluated post-surgery or longitudinally during patient follow-up have been associated with the risk of relapse and prognosis [7], representing a promising biomarker for the evaluation of minimal residual disease. Circulating microRNA (miRNAs) have also been studied as potential biomarkers, as they are very stable in the bloodstream. Several studies have demonstrated that different miRNA profiles are associated with prognosis [5, 8]. Nonetheless, very heterogeneous results emerged from the different studies, which could be mainly due to several factors: the use of serum or plasma, the evaluation of the free counterpart of miRNAs or that encapsulated in extracellular vesicles (EVs), the methodologies used for miRNA normalization, the applied statistical approach. Moreover, the improvement of the different biomarkers with respect to the already recognized clinical parameters (such as pathological stage performance status) was rarely evaluated.

The main objectives of this study were: *i)* to investigate the prognostic potential of circulating cell-free (CF) and extracellular-vesicle (EV)-derived miRNAs in a cohort of surgically treated ES-NSCLC patients, *ii)* to preliminary assess their predictive accuracy, and *iii)* to investigate their added value as compared to basic prognostic factors.

## Materials and methods

### Study design

An international, multicenter, prospective, observational study was conducted to reach the study’s aims. In particular, four centers from three European countries were involved (Italy: IRST-IRCCS, Meldola, and S. Orsola-Malpighi Hospital, Bologna; Spain: Vall d’Hebron hospital/VHIO, Barcelona; France: CHU de Strasburg, Strasburg), and the study protocol was approved by their respective independent ethics committees (IRST-IRCCS: CEROM N.0003097/2018 of 24/04/2018; S.Orsola-Malpighi Hospital: AVEC N.704/2018/OSS/AUSLBO; Hospital Vall d’Hebron/VHIO: PR(AG)188/2018; CPP Ile-de-France, Protocole 22–2019; CHU De Strasburg). The study was conducted following the Declaration of Helsinki, the International Council for Harmonization, good clinical practices, and the applicable legislation on non-interventional observational studies.

Adult patients (> = 18 years of age) diagnosed with a histologically confirmed stage IA-IIIA NSCLC based on the 8th edition TNM classification for lung and pleural tumors [9] surgically resected between November 2018 and December 2020 were included in the study. Patients with concomitant cancer at the time of the ES-NSCLC diagnosis or in the previous 5 years were excluded. A pre-surgery peripheral blood sample was collected (within 3 weeks by the date of surgery). In particular, n. 2 × 10 ml Cell-Free DNA BCT® Streck tubes were collected and centrifuged for plasma obtaining by using specific Standard Operative Procedures (SOPs) shared by the different centers. Specifically, tubes were centrifuged at 1600 × g for 10 min at room temperature, with low acceleration and deceleration, and the upper plasma layer was transferred into a clean conical 15 ml Falcon tube and then centrifuged at 2000 × g for 10 min, at room temperature, with low acceleration and deceleration Plasma was stored at -80 °C until the start of molecular analyses. Demographic, clinical, and treatment information were collected from patient’s medical records and transcribed on the electronic case report forms (eCRFs) created by using the OpenClinica system.

### Isolation of extracellular vesicles and miRNA extraction

Isolation of EVs and associated RNA content was performed as previously described [10]. EVs were also characterized according to MISEV guidelines [10, 11]. Briefly, for each patient, 2 ml of plasma samples were sequentially separated in two distinct fractions of all-sizes vesicular RNAs and circulating cell-free RNAs, including miRNAs, by an all-in-one purification kit based on spin column chromatography with silicon-carbide resin separation matrix (Cat. 56,900, Norgen Biotek Corp, Ontario, Canada). The extracted RNA was eluted in 50 µL and checked on RNA6000 pico chips using the bioanalyzer 2100 instrument (Agilent Technologies, Milan, Italy). Patient EVs were then characterized for size, plasma concentration, and polydispersity data, by using the NanoSight NS300 tracking system (Malvern Instruments Limited, Cambridge, UK). Samples were diluted in PBS to a final volume of 1 mL and concentration, according to the manufacturer’s software manual, within the particles/frame range of 20–120 and the total track to valid track ratio of less than 5 (NanoSight NS300 User Manual, MAN0541-01-EN-00, 2017). Flow cytometry characterization of 37 EV-specific surface markers was done by the MACSPlex Exosome Kit, human (Miltenyi Biotec B.V. & CO. KG, Bergisch Gladbach, Germany), as previously detailed [10]. Samples were diluted with MACSPlex buffer (MPB) to a final volume of 120 μL, 15 μL of MACSPlex Exosome Capture Beads, and 5 uL of each of MACSPlex Exosome Detection Reagent antibodies for ubiquitous EV marker controls (CD9, CD63, CD81) were added to each well. Following buffer incubation and washing steps, flow cytometric analysis was performed with a BD FACSVantage ™ cytofluorimetric (BD Biosciences, Franklin Lakes, NJ, USA). Approximately 10,000 events were recorded per sample. Median fluorescence intensity (MFI) for all 39 capture bead subsets (37 markers plus two background controls) was background corrected (Supplementary Fig. 1). Samples were finally stored at -80 °C until miRNA library preparation. An independent 1 ml plasma aliquot was used to purify intact EVs for nanoparticle characterization.

### miRNA profiling

Separate miRNA libraries were prepared for either CF-miRNAs or EV-RNAs using the Qiaseq miRNA library kit (Qiagen, Milan, Italy). Libraries were prepared following the manufacturer's instructions with some adaptations for low RNA inputs, as previously described. Specifically, higher dilution of ligation adapters and RT primers were adopted to reduce adapter dimers formation; PCR cycles were increased (22 cycles), and an additional bead-based purification step was added to remove unwanted small fragments (< 100 bp). Qubit dsDNA HS assay kit (ThermoFisher, Waltham, MA, USA) and DNA high-sensitivity chips on the Bioanalyzer 2100 instrument (Agilent Technologies, Milan, Italy) were used for library quantification and quality checks. Libraries were then normalized and sequenced on the Nextseq550 instrument (Illumina, San Diego, CA, USA).

### Bioinformatic analysis

The bioinformatics analysis was developed both in the pre-alignment and alignment steps, followed by the identification of microRNAs. The bioinformatic analysis was developed in two steps. All individual steps and the related tools are shown in Supplementary Fig. 2. In the first phase, the bcl files generated by the sequencer were demultiplexed to generate fastq files. Subsequently, a quality assessment of the reads was performed to ensure the goodness of the subsequent analysis. The next step was identifying the UMIs (Unique Molecular Identifiers) within the reads entered during library synthesis. These molecular tags allow counting the initial small RNA molecules in the starting material and reducing possible biases introduced in the PCR amplification step. As the length of mature miRNAs is known to be around 22–25 bp, reads were subsequently cut to remove reads that were too short (< 18 bp) and too long (> 30 bp). These two steps are essential for the next stage of microRNA identification and counting. We then aligned the reads against the mature miRNA sequence database, miRBase version 22, and generated the count table for subsequent analyses.

### Statistical analysis

Data were summarized as mean ± standard deviation (SD), median, and first (IQ) and third (IIIQ) quartiles, as appropriate, for continuous variables and as counts and percentages for categorical variables. The endpoint was disease-free survival (DFS), defined as the time from the date of surgery until the date of disease relapse or death from any cause, whichever occurred first. Patients not experiencing any event were censored at the date of the most recent contact (the last follow-up update was performed on March 31st, 2022).

The Kaplan–Meier (KM) method and log-rank test were used to compare DFS curves between patient groups defined by covariates’ levels. Univariable Cox regression models were used to evaluate the direction and magnitude of the association between covariates and the DFS. Results were reported as hazard ratios (HRs) and median DFS, with corresponding 95% confidence intervals (CIs). Median follow-up time was computed by means of the reverse K-M method. For some analyses, the categories of covariates were grouped due to their low frequency.

Before the mainstream statistical analysis, miRNAs were filtered starting from the raw counts matrix; miRNAs with a third quartile value of fewer than five counts were excluded. Furthermore, samples with a median or first quartile value computed across the miRNAs equal to zero were excluded. Once the miRNAs and samples were filtered out, data were normalized and transformed using the Trimmed Means or M-values ​​(TMM) method and the Blom transformation, respectively. These steps were carried out separately for cell-free and extracellular vesicle miRNAs.

The primary study objectives were to investigate: *i*) the prognostic potential of the miRNAs alone or in combination with standard prognostic factors such as age at surgery, sex, and pathological stage; *ii*) their predictive capacity, and *iii)* their added value in terms of predictive accuracy as compared to standard prognostic factors alone.

To reach these aims, three distinct models were specified: one using the miRNA data only (Model 1), one using both miRNAs and standard prognostic factors (Model 2), and one using the prognostic factors alone (Model 3). Regarding the first two models, given the large number of biomarkers, elastic net regularized Cox regression was used (fixing the mixing parameter to 0.9 and using 5-fold cross-validation—CV—for the identification of the optimal regularization tuning parameter, lambda) and the results reported in terms of beta regression coefficients, as the computation of coefficients’ standard errors as well as other quantities (e.g., confidence intervals) within the context of Cox elastic net models is still a problematic issue. For the third model, the standard Cox model was applied. Given the relatively limited number of observations and events, the predictive accuracy of the survival models was evaluated through a 5-fold CV and based on two metrics: the cross-validated Kaplan–Meier curves and the cross-validated time-dependent Receiver Operating Characteristics (ROC) curves. The CV Kaplan–Meier curves should reflect the ability of the survival model to classify patients characterized by a different risk of relapse or death. The number of groups and how to assign patients to each group should be defined in advance. In our study, we decided to consider only two groups and to assign patients to a group or another based on the median value of the prognostic index (given by the linear predictor) computed using the regression coefficients obtained from a model developed on the training portion of the CV process [12]. The aforementioned prognostic index was used to calculate the CV ROC curves and the corresponding area under the curve (AUC), defining a landmark timepoint of 24 months. Although the log-rank test is usually used to compare KM curves, in the case of CV curves, it is necessary to resort to permutations to obtain the distribution of the test statistic under the null hypothesis and, therefore, evaluate the statistical significance of the log-rank.

Similarly, a permutation-based test must be performed to test the null hypothesis of an AUC of 0.5. To this end, we proceeded by randomly permuting the correspondence between the disease-free survival times, the censoring indicator, and the clinical covariates and miRNAs and repeating the CV procedure for each permutation. The number of permutations was set equal to 500. To evaluate the added value, in terms of predictive accuracy, of the considered biomarkers with respect to basic clinical factors, we proceeded by testing the difference in the log-rank and AUC values between the combined model (miRNA + known prognostic factors) and the model with known prognostic factors only. Also, in this case, the level of significance was determined by resorting to permutations even if, in this case, only the miRNA vector was permuted. The above-mentioned analysis plan was first carried out taking the two sets of miRNAs separately and then together.

The association between demographic and clinical covariates and miRNAs was tested by means of the Student t-test, and *p*-values were adjusted using the Benjamini–Hochberg method.

The statistical analyses were performed using STATA 15.0 (College Station, Texas, USA) and R version 4.2.0, (R Core Team, Vienna, Austria) using mainly the following packages: edgeR, survival, glmnet. The R package *gplots were* used for the graphical representation of selected miRNAs through a heatmap (a hierarchical clustering considering the Euclidean distance measure and the complete agglomeration method was used); the plot was annotated and ordered using the DFS status at 24 months.

### Enrichment analysis

Pathway enrichment analysis of genes targeted by relevant microRNAs was performed using mirNET (https://www.mirnet.ca) [13]. Briefly, miRTarBase v8.0 and TarBase v8.0 were adopted by the software to select microRNA-gene interactions validated experimentally. Then, a network was created, and the minimum network filtering approach was used to reduce network size and keep the main connection patterns. Reactome was then interrogated for pathway enrichment of the network.

Pathway enrichment was performed starting from a single list of CF- and EV- miRNAs associated with DFS from the mainstream statistical analyses (Model 1) and considering separate lists for each type of miRNA. Additional analyses were carried out according to the sign of the miRNA’s relationship with the DFS and the sign of the models’ regression coefficients. Moreover, all analyses were repeated, adding miRNAs correlated (with a Pearson’s correlation coefficient of at least 0.6 in absolute value) with those selected by the mainstream analyses.

## Results

### Demographic and clinical characteristics

A total of 222 patients were enrolled in the study. One hundred and thirty-three patients were males (59.9%), the median age at surgery was 70 years (IQ-IIIQ: 63–75), and the majority (86.1%) had a history of smoking, Table 1. In addition, most patients (95.8%) had an ECOG performance status (PS) of less than or equal to 1. One hundred and thirty-seven patients (64.3%) had a pathologic stage I tumor, 36 (16.9%) a stage II tumor, and 40 (18.8%) a stage IIIA cancer. Adenocarcinoma was the most common histologic type (81.0%). Twenty-one (9.5%) patients received a pre-surgery neo-adjuvant therapy, whereas 31 (14.7%) and 14 (6.6%) patients received adjuvant chemo- and/or radiotherapy, respectively.

**Table 1:** Baseline patient characteristics

	**n**	**%**
**Gender**		
F	89	40.1
M	133	59.9
**Age at surgery (yrs)**		
Mean ± SD	69 ± 8	
Median [IQ—IIIQ]	70 [63−75]	
Min—max	44 – 85	
*missing*	1	
**Smoking habit**		
Never smoker	29	13.9
Ex-smoker	128	61.2
Current smoker	52	24.9
*missing*	13	
**ECOG PS**		
0	149	69.3
1	57	26.5
2	9	4.2
*missing*	*7*	
**Neo-adjuvant therapy**		
No	200	90.5
Yes	21	9.5
*missing*	*1*	
**Type of resection**		
Atypical resection	10	9.1
Pneumectomy	8	3.6
Lobectomy	193	87.3
*missing*	*1*	
**Radicality**		
R0	212	96.8
R1	6	2.7
R2	1	0.5
*missing*	*3*	
**Histological diagnosis**		
Adenocarcinoma	179	81.0
Squamous cell carcinoma	42	19.0
*missing*	*1*	
**Pathological stage**		
IA	99	46.5
IB	38	17.8
IIA	9	4.2
IIB	27	12.7
IIIA	40	18.8
*missing*	*9*	
**Grading**		
G1	23	13.1
G2	112	64.0
G3	40	22.9
*missing*	*47*	

Percentages may not equal 100 due to rounding

*SD* standard deviation, *IQ* first quartile, *IIIQ* third quartile, *ECOG* Eastern Cooperative Oncology Group, *PS* performance status

### MiRNAs and demographic and clinical covariates

Supplementary Fig. 3 shows the patients’ disposal for the main statistical analyses of the study. The prognostic role of the biomarkers was first investigated for the CF-miRNAs and EV-miRNAs separately and then together. The unsupervised filtering of patients and miRNAs was done separately once and for the two types of miRNAs. Thus, the patients’ biological samples that passed the quality check were 176 and 171, whereas the retained miRNAs were 438 and 232 for CF- and EV-miRNAs.

The association analysis between the normalized CF-miRNAs and clinical information showed that the expression of miR-224-5p and miR-651-5p was significantly lower in males than in females (adjusted *p*-values < 0.001 and 0.002, respectively), as well as that of miR-877-3p and miR-3617-5p with regard to higher ECOG PS values (adjusted *p*-values < 0.041 and 0.044, respectively). MiR-185-3p was highly expressed in squamous-cell histotype compared to adenocarcinomas (adjusted *p*-value = 0.017) (Supplementary Fig. 4 Panel A). No other significant associations were observed. Concerning EV-miR, two of them – miR-224-5p and miR-143-3p – showed significantly lower expression values among males than females (adjusted *p*-values < 0.001 and 0.016, respectively) (Supplementary Fig. 4 Panel B). Seven EV-miR were associated with the pathological stage (miR-431-5p, miR-487b-3p, miR-9.5p, miR-654-3p, miR-369-3p, miR-323a-3p, miR-376c-3p). In particular, all except one (miR-9-5p) showed decreasing expression values with increasing stage (Supplementary Fig. 4 Panel B).

### Prognostic potential of CF- and EV-miRNAs

The information on DFS was available for 195 (88%) patients. The median follow-up time was 26.3 months (95% CI: 25.4—27.6), and the median DFS was not reached (NR). A total of 52 events were observed. Table 2 shows the results obtained by fitting three distinct models, as described in the Statistical Analysis section. Models 1 and 2 were obtained using elastic-net penalized Cox regression, whereas Model 3 by means of standard Cox regression.

**Table 2:** Multivariable Cox proportional hazards regression models on disease-free survival (separate analyses for CF- and EV-miRNA)

	**Model 1**	**Model 2**	**Model 3**		
	**Coef**	**Coef**	**Coef**	**HR (95% CI)**	***p*-value**
**CF-miRNA**					
miR-135a-5p	-0.005				
miR-29c-3p	0.083	0.056			
miR-877-3p	-0.243	-0.180			
Sex (M vs F)		0.265	0.295	1.34 (0.69–2.60)	0.381
Age at surgery		0.025	0.027	1.03 (0.98–1.07)	0.216
pSTAGE (II vs I)		1.032	1.013	2.75 (1.28–5.91)	0.009
pSTAGE (IIIA vs I)		1.389	1.013	4.23 (2.09–8.53)	< 0.001
**EV-miRNA**					
miR-127-3p	-0.089				
miR-1277-3p	-0.223				
miR-136-3p	-0.182				
miR-181a-2-3p	-0.148	-0.053			
miR-182-5p	0.089	0.032			
miR-18a-5p	-0.018				
miR-192-5p	0.208	0.185			
miR-32-5p	0.067				
miR-323b-3p	-0.030				
miR-328-3p	-0.039				
miR-339-3p	0.139				
miR-361-3p	0.034				
miR-3615	-0.208				
miR-370-3p	-0.122				
miR-5187-5p	0.211				
miR-532-3p	-0.200	-0.113			
miR-589-5p	-0.054	-0.008			
miR-628-5p	-0.039				
miR-6852-5p	-0.018				
miR9-5p	0.039				
miR-99b-5p	-0.109				
Sex (M vs F)		0.115	0.178	1.20 (0.61–2.32)	0.599
Age at surgery		0.008	0.011	1.01 (0.97–1.06)	0.611
pSTAGE (II vs I)		0.988	0.933	2.54 (1.16–5.54)	0.019
pSTAGE (IIIA vs I)		1.566	1.494	4.46 (2.18–9.10)	< 0.001

Model 1 refers to the results obtained fitting an elastic net penalized Cox model on the microRNA data only; Model 2 refers to the results obtained fitting an elastic net penalized Cox model on the microRNA and basic prognostic factors data; Model 3 refers to the results obtained fitting a standard Cox model only on the data of basic prognostic factors

*CF* cell-free, *EV* extracellular vesicle, *HR* hazard ratio, *CI* confidence intervals, *pSTAGE* pathologic disease stage, *Coef* beta regression coefficient

**Fig. 1:**
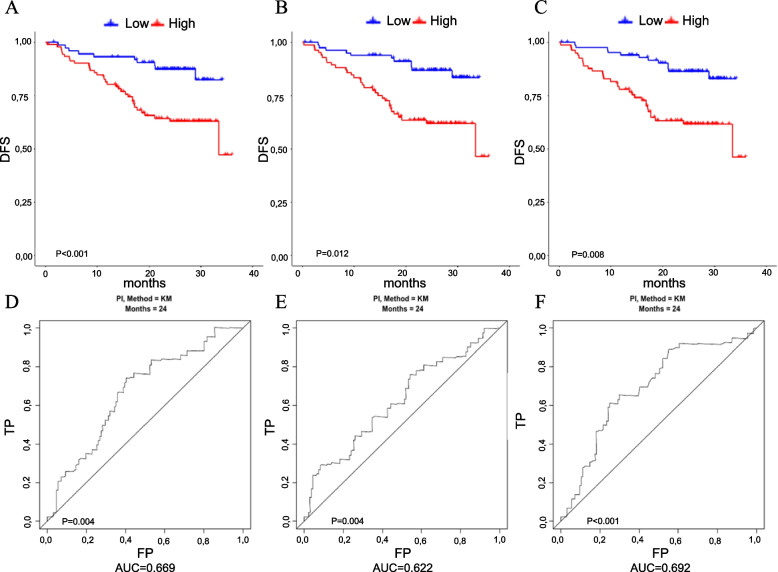
Performance of the models using cell-free miRNA data. From (**A**) to (**C**) 5-fold cross-validated Kaplan–Meier curves for the models derived using the miRNA data alone, miRNA and and basic prognostic factors (age, sex, and pathologic stage) data, and the prognostic factors data alone. From (**D**) to (**F**) Five-fold cross-validated time-dependent ROC curves (at 24 months) for the three models. *DFS* disease-free survival, *TP* true positive, *FP* false positive

**Fig. 2:**
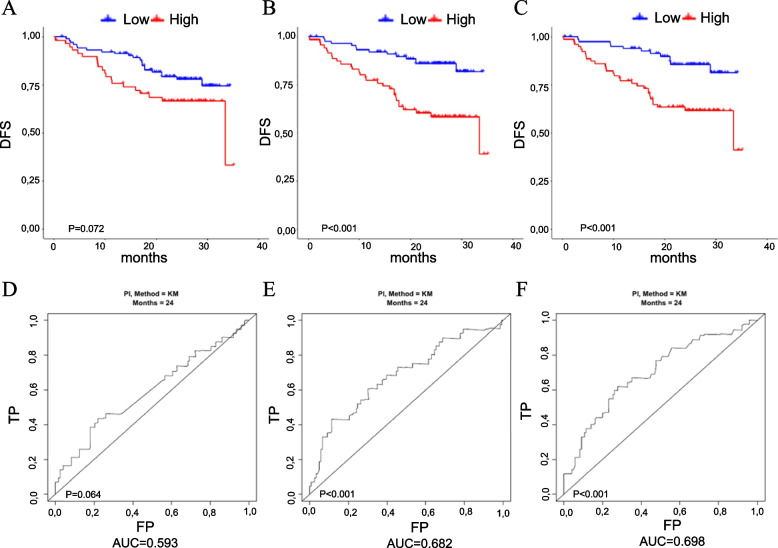
**P**erformance of the models using extracellular vesicle miRNA data. From (**A**) to (**C**) 5-fold cross-validated Kaplan–Meier curves for the models derived using the miRNA data alone, miRNA and basic prognostic factors (age, sex, and pathologic stage) data, and prognostic factors data alone. From (**D**) to (**F**) Five-fold cross-validated time-dependent ROC curves (at 24 months) for the three models. *DFS* disease-free survival, *TP* true positive, *FP* false positive

Model 1 aimed at evaluating the prognostic potential of the miRNAs alone. From this analysis, three CF-miRNAs and 21 EV-miRNAs were associated with DFS (had beta coefficients different from zero). Most of them reported a negative regression coefficient indicating that as the expression of the miRNA increases, the hazard of relapse or death decreases. The performance of this model is reported in Fig. 1, panels A and D, for the analyses on cell-free miRNAs, and in Fig. 2, panels A and D, for those on extracellular derived miRNAs. The model derived from the CF-miRNA data showed, as compared to the models derived from the EV-miRNAs, better performance in terms of both CV Kaplan–Meier curves separation and discriminatory accuracy (AUCs were 0.67 and 0.59, respectively).

The second and third models were fitted especially to investigate whether the miRNA data added predictive accuracy to a model, including readily available information such as age, sex, and stage of disease. The second model was derived from the miRNA, demographic, and clinical data. In this model, two CF-miRNAs (miR-29c-3p and miR-877-3p) and five EV-miRNAs (miR-181a-2-3p, miR-182-5p, miR-192-5p, miR-532-3p and miR-589-5p) remained associated with the DFS. In particular, higher expression of the cell-free miR-29c-3p and the two extracellular vesicle miRNAs, miR-182-5p and miR-192-5p, was associated with a worse prognosis. Conversely, a higher expression of the cell-free miR-877-3p and of the extracellular vesicle miRNAs, miR-181a-2-3p, miR-532-3p, and miR-589-5p, was associated with a better prognosis. The performance of these combined models was almost comparable in terms of curve separation but slightly better for the model including extracellular derived miRNAs in terms of AUC, equals to 0.68 as compared to a value of 0.62 obtained from the combined model including cell-free miRNAs, Figs. 1 and 2, panels B and E.

The third model included age at surgery, sex, and the pathologic stage of disease alone and was fitted to investigate if the molecular information can significantly improve the predictive accuracy of known prognostic factors routinely available in clinical practice. Age at surgery was included as a 1-unit increment continuous covariate, and the stage was grouped into three main categories. Table 2 reports two hazard ratios for each of these factors as they were estimated in the two separate datasets, one with CF-miRNA data and one with EV-miRNA data that included a slightly different number of patients due to the filtering procedures described in the Statistical Analysis section and cited above.

Supplementary Table 1 shows the results of univariate Cox models fitted on the 195 patients with available DFS data, whereas Supplementary Fig. 5 the DFS curves for each stage category. Given the small number of patients within each subtype of stages I, II, and III, in Model 3 reported in Table 2, only the three main categories for the stage were considered.

Thus, the model including only the clinical factors, Model 3, showed per se good predictive performance, as shown in Figs. 1 and 2, panels C and F.

The combined models (one for CF- and one for EV-miRNAs), containing both miRNAs and clinical factors (age, sex, stage), did not show significantly higher performance compared to the model including the non-biological factors alone (Models 2 *vs*. Models 3). From the comparison of the CV Kaplan–Meier curves derived from these models (Fig. 1, panels B and C for CF and Fig. 2, panels B and C for EV), and of the AUCs of the CV ROC curves it emerges that miRNAs do not provide additional survival risk discrimination to that already provided by basic covariates (the permuted log-rank *p*-value for the comparison of the two models was equal to 0.640 and 0.248 for CF- and EV-miRNA, respectively, whereas the *p*-value comparing the AUCs was equal to 0.076 and 0.652 for CF- and EV-miRNA, respectively,).

Repeating the analysis, considering the two types of miRNAs together (CF- and EV-derived), we obtained the results shown in Supplementary Table 2. These models were fitted on 156 patients (41 events), as reported in Figure S6. Model 1 included 14 miRNAs, most of them, 9, already found associated with DFS in the separate analyses described above. Based on this model, modestly separated survival risk groups were obtained (permuted log-rank *p*-value = 0.061) and an AUC of 0.62 (permuted *p*-value = 0.021), Supplementary Fig. 6 panel A. Model 2 included three CF- (miR-135a-5p, miR-29c-3p, miR-877-3p) and one EV-miRNA (miR-192-5p), other than standard prognostic factors. Its performance was better than the previous model, Supplementary Fig. 6 panel B. Indeed, it showed a better separation of the survival curves (permuted log-rank *p*-value < 0.001) as well as AUC (0.64, permuted *p*-value < 0.001). Compared to the model including the standard prognostic factors alone, Model 3 and Model 2 showed better survival curve separation (permuted log-rank *p*-value = 0.016) and a comparable discriminatory capacity (permuted *p*-value per AUCs comparison = 0.960).

From analogous subgroup analyses on stage I patients, 10 CF-miR were associated with DFS. For most of them (miR-135a-5p, miR-877-5p, miR-107, miR-1226-3p, miR-362-5p, miR-3913-5p, miR-548ax), increasing expression values associated with a reduced hazard of relapse or death whereas for miR-345-5p, miR-9-3p, the opposite was observed. When considering also the standard prognostic factors, four CF-miR were retained in the model (miR-135a-5p, miR-107, miR-548ax, and miR-9-3p); the direction of the association was the same as before. The models’ performance was not satisfactory and there was no evidence of an incremental predictive capacity compared to age and sex alone, results not shown. This is perhaps due to the small size and the immature follow-up length for this subgroup of patients. No EV-miR was found to predict DFS in this population.

### Pathway enrichment

**Fig. 3:**
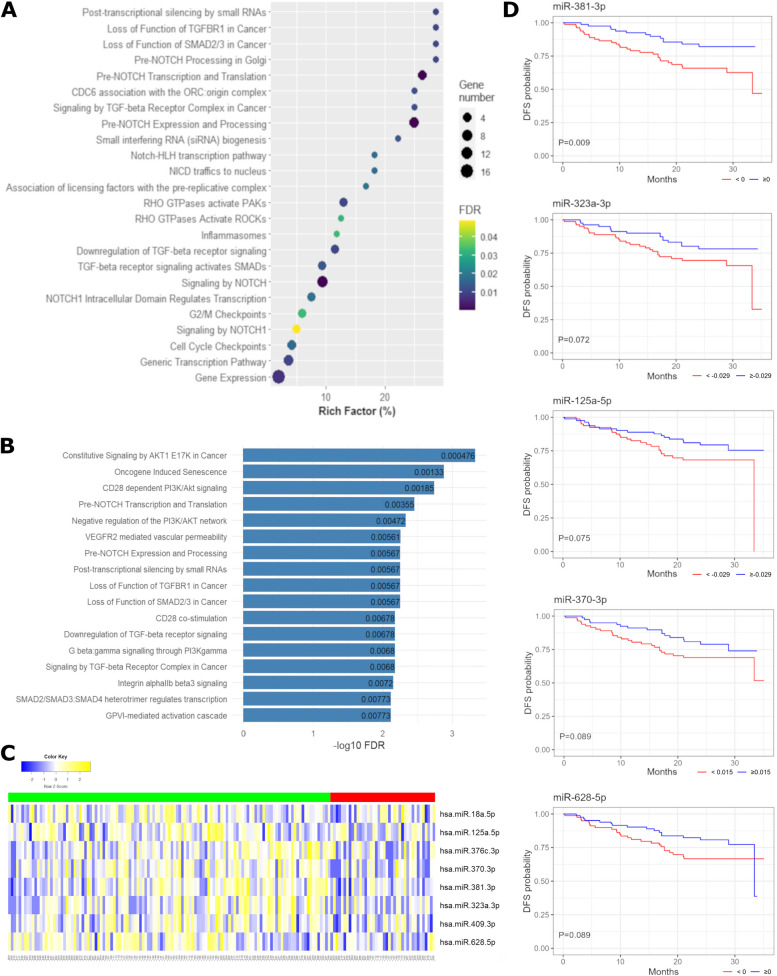
Pathway enrichment. **A** Bubble plot showing the most enriched pathways of 24 miRNAs (3 CF-miRNAs and the 21 EV-miRNAs) associated with disease-free survival. **B** Most significant enriched pathways targeted by EV-miRNAs under expressed in patients with worse outcome **C** Heatmap showing normalized expression levels of the 8 enriched miRNAs targeting TGF-beta pathways. Patients alive and not relapsed within 24 months are shown in green (on the right) whereas patients encountering disease relapse or death within 24 months are indicated in red (on the left). **D**) Kaplan Meier curves for disease-free survival for each miRNA

Pathway enrichment was first conducted by analyzing the 3 CF-miRNAs and the 21 EV-miRNAs associated with DFS from the mainstream statistical analyses (Model 1). Interestingly, TGF-beta and NOTCH were the most enriched among the significant pathways found (FDR < 0.05). In addition, at a lower rich factor, involvement in the cell cycle and immune regulation could be identified (Fig. 3A). To further dissect the role of miRNA specifically involved in these pathways, we deepened the enrichment analysis using an expanded list of miRNAs, considering also those moderately to highly correlated with the one listed above (the list of correlated miRNAs is reported in Supplementary Table 3). Pathway enrichment, with or without including correlated miRNAs, demonstrated only minor changes (data not shown). However, it significantly improved the enrichment in the subsequent analysis, wherein the original list of miRNAs was further divided into additional subtypes.

Then, we evaluated separately the pathways that resulted in being enriched by miRNAs derived from CF and those derived from EV. In particular, considering both miRNAs deriving from the mainstream analysis and their correlation, we found that miRNAs involved in regulating genes participating in TGF-beta and NOTCH pathways were enriched in the EV fraction. In addition, miRNAs targeting components of tyrosine kinases and of PI3K signalling pathway can be found enriched in EV. Conversely, the miRNAs identified in the CF fraction were predominantly involved in more comprehensive pathways, such as the cell cycle and immune system (Supplementary Tables 4–5).

Thus, an additional analysis focusing on the EV fraction was performed. That is, considering the EV-miRNAs with positive (higher expression values associated with a shorter DFS or higher instantaneous hazard of relapse or death) and negative regression coefficients (higher expression values associated with a longer DFS or lower instantaneous hazard of relapse or death) separately. For this step, positively correlated miRNAs were added to each miRNA list before enrichment analysis. MiRNAs with positive regression coefficients were shown to target genes involved in vesicle transport, mitotic activities, and the immune system. On the other hand, miRNAs with negative regression coefficients were found to have a stronger association with TGF-beta/SMAD2/3 and NOTCH pathways (Fig. 3C, Supplementary Tables 6–7). In particular, 8 miRNAs were involved in the enrichment of these pathways (miR-18a-5p, miR-370-3p, miR-628-5p, miR-125a-5p, mir376c-3p, miR-381-3p, miR323a-3p, miR-409-3p) through the targeting of TGFBR2 and SMAD2. Accordingly, the expression level of these 8 miRNAs was globally lower in patients with worse outcomes (Fig. 3C); in particular, 5 of these (miR-125a-5p, miR-370-3p, miR-628-5p, miR-381-3p, miR323a-3p) were associated with DFS (Fig. 3D).

## Discussion

Circulating miRNAs have attracted much interest in cancer diagnosis and prognosis as they are small and very stable in circulation and might have essential functions. For these reasons, they are good candidates as molecular biomarkers capable of selecting patients at higher risk of recurrence. However, very conflicting results regarding their role have been reported, leaving doubts about their real value as prognostic markers. The heterogeneous results are mainly due to different aspects: the panel of miRNAs analyzed (small panel of miRNAs or miRNome analysis), the methodology used for miRNA evaluation and normalization, the statistical approach applied, the study endpoint, the specimen used for the analysis (plasma or serum) and methods of collection. Moreover, “circulating miRNAs” can be different whether we consider EV-derived miRNAs or the overall circulating miRNAs content, as it is well established that EV-derived miRNAs could exert more specific functions in the context of lung cancer, with respect to those released in the circulating compartment [14, 15]. Furthermore, despite numerous studies that have analyzed miRNAs as potential prognostic factors, it is not clear the added value of miRNAs with respect to the established prognostic factors used in the clinic, mainly the stage of the disease.

To address some of these aspects, we performed a prospective multicentre study to clarify the role of circulating miRNAs as prognostic biomarkers in ES-NSCLC, focusing on the added value of the miRNAs with respect to the known clinicopathological prognostic parameters.

From the model built on the miRNA data, we found that 3 CF-miRNAs and 21 EV-miRNAs were associated with DFS without considering the integration of known clinical prognostic factors. Most of them exhibited a negative regression coefficient, meaning that their higher expression values are associated with a better prognosis, pointing out a tumor-suppressor function of the specific miRNAs.

Concerning the CF-miRNAs, miR-135a-5p, and miR-877-3p were overexpressed in patients with a lower risk of relapse or death, which aligns with their reported biological function. It was shown that miR-877 overexpression repressed NSCLC cell growth by targeting tartrate-resistant acid phosphatase (TRAP), also known as acid phosphatase 5 (ACP5), and inhibiting the PI3K/AKT pathway [16]. Concerning miR-135a-5p, it has been shown in several studies that its expression decreased in different solid tumors such as glioma [17], gallbladder [18], colorectal cancer [19], and also lung cancer [20]. Conversely, we observed that CF miR-29c-3p upregulation was associated with worse DFS. This observation goes along with its potential role as a tumor promoter, recently shown in ovarian cancer models [21].

On the other hand, we found 21 EV-derived miRNAs significantly associated with patient prognosis. Of these, 12 (miR-127-3p, miR-1277-3p, miR-136-3p, miR-181a-2-3p, miR-18a-5p, miR-323b-3p, miR-3615, miR-532-3p, miR-589-5p, miR-628-5p, miR-6852-5p and miR-99b-5p) might act as tumor suppressors, with an association between high levels and a better prognosis. The demonstrated biological role of most of these miRNAs aligns with our results. In particular, it has been shown that miR-181a-2-3p is downregulated in NSCLC tissue compared with control samples and that its low expression in tumor tissue and plasma is associated with longer progression-free survival [22]. Interestingly, plasma EV-derived miR-3615, combined in a model with other 3 miRNAs, was demonstrated to be able to distinguish early-stage lung adenocarcinoma patients with respect to healthy donors, suggesting a potential role as a noninvasive biomarker for the early detection [23]. A tumor suppressor activity was also demonstrated for miR-532-3p that, by targeting FOXP3, can inhibit the growth of NSCLC cell models [24] and for miR-589-5p, which, since it usually inhibits Histone deacetylases 5 (HDAC5), when downregulated leads to an overexpression of HDAC5 and therefore to an increase in cancer cell proliferation [25]. Concerning miR-18a-5p, its complex role in cancer has been demonstrated, as it can act both as an oncogene and a suppressor [26]. Among the 9 EV-derived miRNAs whose overexpression resulted associated with shorter DFS (miR-182-5p, miR-192-5p, miR-32-5p, miR-328-3p, miR-339-3p, miR-361-3p, miR-370-3p, miR-5187-5p, and miR-9-5p), suggesting their role as oncomiR, their demonstrated biological role in the literature is in part in line and in part in contrast with our results. In particular, a role as oncomiR has been confirmed for miR-182-5p [27], miR-328-3p [28], miR-339-3p [29], miR-361-3p [30], and miR-9-5p [31]. Conversely, contrasting results are reported regarding miR-192-5p [32], miR-370-3p [33], and miR-32-5p [34], as from literature results, they seem to have a role as tumor suppressor miRNAs.

Cell-free miRNAs can be released and uptaken by cells through vesicle trafficking and protein carrier mechanisms, and they are able to function as gene expression regulators in cell to-cell communication mechanisms under normal and pathological conditions, such as cancer [35]. However, we have to consider that miRNAs are released into the bloodstream from different cell types, not only from tumor cells [36], and this still unclear aspect leaves some open questions regarding the real role that these miRNAs found in the blood circulation may have. Moreover, miRNAs can target several different genes and as a consequence can affect different pathways. Taking this into consideration, a pathway enrichment analysis was performed to reach a global overview of the different pathways on which the significant miRNAs are involved.

Interestingly, enrichment of TGF-beta/SMAD and NOTCH pathways was observed, which was even more evident when considering only EV-derived miRNAs. In particular, 5 miRNAs (miR-125a-5p, miR-370-3p, miR-628-5p, miR-381-3p, miR323a-3p), which evidence of interaction with the TGF-beta pathway is present in the literature [37–41], were found to have higher expression levels in patients with a longer DFS in our cohort.

TGF-beta/SMAD and NOTCH are well-known prognostic pathways in NSCLC [42–45]. In particular, a specific association between TGF-beta expression and risk of relapse in ES-NSCLC has been shown [46]. With regard to NOTCH, a previous study showed that specific polymorphisms of the gene are associated with survival rates in ES-NSCLC [47]. Moreover, an in vitro study demonstrated that the NOTCH signaling significantly affects the growth and the malignant phenotype of both colorectal and lung models [48]. Interestingly, an enrichment of the PI3K pathway was also observed in EV-derived miRNAs. The PI3K pathway involvement in the prognostic risk determination of ES-NSCLC was already shown in previous studies [49, 50], in accordance with our results. Overall, we found an evident enrichment of TGF-beta/SMAD, NOTCH, and PI3K pathways in EV-derived miRNAs.

This reinforce the already demonstrated role of EVs as components with specific functional activities in the regulation of cell growth, which consequently can have important prognostic and predictive roles in response to therapies [51]. It has been shown that both tumor and immune cells can release specific EVs containing components, mainly miRNAs, with specific functions able to regulate cancer-specific processes such as epithelial-mesenchymal transition [52, 53], neovascularization [54, 55], anti-tumor immune cell function [56, 57]. Hence, we can conclude that EV-derived miRNAs seem to have more specific functions with respect to CF-miRNAs, which could be derived by EV itself but also from other, more non-specific sources, such as necrotic or apoptotic cells.

To evaluate the added predictive value of the miRNAs to basic prognostic factors, statistical models combining these and disease stage, age, and sex were specified and then compared with a model including only the demographic and clinical factors. In the combined models (one for CF- and one for EV-miRNAs), two CF-miRNAs (miR-29c-3p and miR-877-3p) and five EV-miRNAs (miR-181a-2-3p, miR-182-5p, miR-192-5p, miR-532-3p and miR-589-5p) remained associated with DFS and we did not observe significantly higher predictive performance compared to the models including the clinical factors alone. However, when we derived the combined model considering the data from both types of miRNAs, a significant increase in the prognostic accuracy was found. In particular, 3 CF-miRNAs (miR-135a-5p, miR-29c-3p, miR-877-3p) and one EV-miRNA (miR-192-5p) gave a substantial contribution in addition to the clinical factors.

Our study has several strengths and limitations. Among the primary strengths are i) the prospective collection of biological specimens and clinical data, ii) the analysis of the miRNome and differentiation of miR origins, iii) Moreover, despite the relatively small sample size and the lack of an external validation cohort, we were still able to preliminarily evaluate the performance of the fitted models, as well as, through the approach proposed by Simon et al. [12], to investigate the added predictive value of the miRNAs as compared to basic non-biological prognostic factors, in particular the stage of disease, something that is not often reported in the published studies.

As mentioned above, one limitation of this study is the modest sample size and the limited follow-up time (the median was about 26 months), especially given the high proportion of stage I cancers in our cohort (64.3%). Both these factors limited the possibility of stratified analyses and their statistical power, leaving several questions unanswered, for example, whether different miRNAs are involved in the prognosis prediction by disease characteristics (e.g., stage and histotype) or if their prognostic effect may vary within strata. However, enrollment is ongoing in another funded study on ES-NSCLC carried out by some of the RESTING centers, which will permit us to validate our data. Another limitation is relating to the uncertain source of release of the miRNAs found in circulation, which leaves some open questions regarding their functional role in cancer development. Moreover, we are conscious that the different methodologies available for EV isolation could maintain a portion of small lipoproteins, that could interfere with the subsequent analyses. However, we used an EV purification method that relies on the specific binding of EV surface proteins to negatively charged silicon membranes, exploiting differences in pH and isoelectric points compared to other proteins like histones and lipoproteins in biological fluids. This approach allows for the selective adsorption and subsequent elution of EV while effectively excluding most lipoprotein contaminants, as demonstrated in existing literature [58]. Given that there are no gold standard procedures for EV isolation, some open questions remain about the optimal methodology to be used. However, our approach seems to be reproducible and robust with some potentiality to be included in the clinical practice in the future.

In summary, our study highlights potential miRNAs able to predict the risk of relapse after surgery in patients with ES-NSCLC. When considered separately, CF- and EV-miRNAs could not significantly improve the prognostic performance of the model with known clinical prognostic factors. However, when considering them together, a significant improvement was observed. Thus, the identified circulating miRNAs could represent a non-invasive approach that could permit clinicians to have further information to decide the most appropriate patient’s management.

### Supplementary Information

Supplementary Material 1

## Data Availability

The raw data generated in this study will be available on European Genome phenome Archive (EGA) at https://ega-archive.org/datasets/ searching for RESTING study.

## References

[1] Sung H, Ferlay J, Siegel RL, et al. Global Cancer Statistics 2020: GLOBOCAN estimates of incidence and mortality worldwide for 36 cancers in 185 countries. CA Cancer J Clin. 2021;71(3):209–49. 33538338 10.3322/caac.21660

[2] Uramoto H, Tanaka F. Recurrence after surgery in patients with NSCLC. Transl Lung Cancer Res. 2013;3(4):242–9. 10.3978/j.issn.2218-6751.2013.12.05. 10.3978/j.issn.2218-6751.2013.12.05 PMC4367696 25806307

[3] Cho WCS, Tan KT, Ma VWS, et al. Targeted next-generation sequencing reveals recurrence-associated genomic alterations in early-stage non-small cell lung cancer. Oncotarget. 2018;9(91):36344–57. 10.18632/oncotarget.26349. 30555633 10.18632/oncotarget.26349 PMC6284742

[4] Wistuba II, Behrens C, Lombardi F, et al. Validation of a proliferation-based expression signature as prognostic marker in early stage lung adenocarcinoma. Clin Cancer Res. 2013;19(22):6261–71. 10.1158/1078-0432.ccr-13-0596. 24048333 10.1158/1078-0432.ccr-13-0596 PMC3834029

[5] Ulivi P, Petracci E, Marisi G, et al. Prognostic role of circulating miRNAs in early-stage non-small cell lung cancer. J Clin Med. 2019;8(2):131. 10.3390/jcm8020131. 30678026 10.3390/jcm8020131 PMC6407000

[6] Kratz JR, Li JZ, Tsui J, et al. Genetic and immunologic features of recurrent stage I lung adenocarcinoma. Sci Rep. 2021;11(1):23690. 10.1038/s41598-021-02946-0. 34880292 10.1038/s41598-021-02946-0 PMC8654957

[7] Qiu B, Guo W, Zhang F, et al. Dynamic recurrence risk and adjuvant chemotherapy benefit prediction by ctDNA in resected NSCLC. Nat Commun. 2021;12(1):6770. 10.1038/s41467-021-27022-z. 34799585 10.1038/s41467-021-27022-z PMC8605017

[8] Baverel PG, Dubois VFS, Jin CY, et al. Population pharmacokinetics of durvalumab in cancer patients and association with longitudinal biomarkers of disease status. Clin Pharmacol Ther. 2018;103(4):631–42. 10.1002/cpt.982. 29243223 10.1002/cpt.982 PMC5887840

[9] Goldstraw P, Chansky K, Crowley J, et al. The IASLC lung cancer staging project: proposals for revision of the TNM stage groupings in the forthcoming (Eighth) Edition of the TNM classification for lung cancer. J Thorac Oncol. 2016;11(1):39–51. 10.1016/j.jtho.2015.09.009. 26762738 10.1016/j.jtho.2015.09.009

[10] Pasini L, Vannini I, Ulivi P, et al. Comparative analysis of free-circulating and vesicle-associated plasma microRNAs of healthy controls and early-stage lung cancer patients. Pharmaceutics. 2022;14(10):2029. 10.3390/pharmaceutics14102029. 36297464 10.3390/pharmaceutics14102029 PMC9610033

[11] Welsh JA, Goberdhan DCI, O’Driscoll L, et al. Minimal information for studies of extracellular vesicles (MISEV2023): from basic to advanced approaches. J Extracell Vesicles. 2024;13(2):e12404. 10.1002/jev2.12404. 38326288 10.1002/jev2.12404 PMC10850029

[12] Simon RM, Subramanian J, Li MC, Menezes S. Using cross-validation to evaluate predictive accuracy of survival risk classifiers based on high-dimensional data. Brief Bioinform. 2011;12(3):203–14. 10.1093/bib/bbr001. 21324971 10.1093/bib/bbr001 PMC3105299

[13] Chang L, Zhou G, Soufan O, Xia J. miRNet 2.0: network-based visual analytics for miRNA functional analysis and systems biology. Nucleic Acids Res. 2020;48(W1):gkaa467. 10.1093/nar/gkaa467. 10.1093/nar/gkaa467 PMC7319552 32484539

[14] Shukuya T, Ghai V, Amann JM, et al. Circulating MicroRNAs and extracellular vesicle-containing MicroRNAs as response biomarkers of anti–programmed cell death protein 1 or programmed death-ligand 1 therapy in NSCLC. J Thorac Oncol. 2020;15(11):1773–81. 10.1016/j.jtho.2020.05.022. 32565389 10.1016/j.jtho.2020.05.022 PMC7641981

[15] Kinoshita T, Yip KW, Spence T, Liu FF. MicroRNAs in extracellular vesicles: potential cancer biomarkers. J Hum Genet. 2017;62(1):67–74. 10.1038/jhg.2016.87. 27383658 10.1038/jhg.2016.87

[16] Bai X, He C, Fu B, et al. microRNA-877 contributes to decreased non-small cell lung cancer cell growth via the PI3K/AKT pathway by targeting tartrate resistant acid phosphatase 5 activity. Cell Cycle. 2020;19(23):3260–76. 10.1080/15384101.2020.1839697. 33222607 10.1080/15384101.2020.1839697 PMC7751652

[17] Luo W, Sun C, Zhou J, et al. miR-135a-5p functions as a glioma proliferation suppressor by targeting tumor necrosis factor receptor-associated factor 5 and predicts patients’ prognosis. Am J Pathol. 2019;189(1):162–76. 10.1016/j.ajpath.2018.08.019. 30312580 10.1016/j.ajpath.2018.08.019

[18] Diao H, Xu X, Zhao B, Yang G. miR-135a-5p inhibits tumor invasion by targeting ANGPT2 in gallbladder cancer. Mol Med Rep. 2021;24(1):528. 10.3892/mmr.2021.12167. 34036386 10.3892/mmr.2021.12167 PMC8170269

[19] Wang J, Yang J, Zhang H, Liao Y, Xu D, Ma S. Effects of miR-135a-5p and miR-141 on proliferation, invasion and apoptosis of colorectal cancer SW620 cells. Oncol Lett. 2020;20(1):914–20. 10.3892/ol.2020.11598. 32566020 10.3892/ol.2020.11598 PMC7286134

[20] Jin X, Guan Y, Zhang Z, Wang H. Microarray data analysis on gene and miRNA expression to identify biomarkers in non-small cell lung cancer. BMC Cancer. 2020;20(1):329. 10.1186/s12885-020-06829-x. 32299382 10.1186/s12885-020-06829-x PMC7164187

[21] Zhao H, Feng L, Cheng R, et al. miR-29c-3p acts as a tumor promoter by regulating β-catenin signaling through suppressing DNMT3A, TET1 and HBP1 in ovarian carcinoma. Cell Signal. 2024;113:110936. 10.1016/j.cellsig.2023.110936. 37925048 10.1016/j.cellsig.2023.110936

[22] Simiene J, Dabkeviciene D, Stanciute D, et al. Potential of miR-181a-5p and miR-630 as clinical biomarkers in NSCLC. BMC Cancer. 2023;23(1):857. 10.1186/s12885-023-11365-5. 37697308 10.1186/s12885-023-11365-5 PMC10496384

[23] Gao S, Guo W, Liu T, et al. Plasma extracellular vesicle microRNA profiling and the identification of a diagnostic signature for stage I lung adenocarcinoma. Cancer Sci. 2022;113(2):648–59. 10.1111/cas.15222. 34837453 10.1111/cas.15222 PMC8819331

[24] Jiang W, Zheng L, Yan Q, Chen L, Wang X. MiR-532-3p inhibits metastasis and proliferation of non-small cell lung cancer by targeting FOXP3. J BU : Off J Balk Union Oncol. 2019;24(6):2287–93. 31983096

[25] Liu C, Lv D, Li M, et al. Hypermethylation of miRNA-589 promoter leads to upregulation of HDAC5 which promotes malignancy in non-small cell lung cancer. Int J Oncol. 2017;50(6):2079–90. 10.3892/ijo.2017.3967. 28440397 10.3892/ijo.2017.3967

[26] Kolenda T, Guglas K, Kopczyńska M, et al. Good or not good: role of miR-18a in cancer biology. Rep Pr Oncol Radiother. 2020;25(5):808–19. 10.1016/j.rpor.2020.07.006. 10.1016/j.rpor.2020.07.006 PMC7451592 32884453

[27] Yang W, Yin Y, Bi L, et al. MiR-182-5p promotes the metastasis and epithelial-mesenchymal transition in non-small cell lung cancer by targeting EPAS1. J Cancer. 2021;12(23):7120–9. 10.7150/jca.60419. 34729113 10.7150/jca.60419 PMC8558643

[28] Ghafouri-Fard S, Safarzadeh A, Katiraei SHF, Hussen BM, Hajiesmaeili M. Diverse functions of miR-328 in the carcinogenesis. Pathol - Res Pr. 2023;251:154896. 10.1016/j.prp.2023.154896. 10.1016/j.prp.2023.154896 37852016

[29] Trakunram K, Chaniad P, Geater SL, et al. Serum miR-339-3p as a potential diagnostic marker for non-small cell lung cancer. Cancer Biol Med. 2020;17(3):652–63. 10.20892/j.issn.2095-3941.2020.0063. 32944397 10.20892/j.issn.2095-3941.2020.0063 PMC7476089

[30] Zhou D, Ji G, Wei G, et al. MiR-361-3p promotes tumorigenesis of osteosarcoma cells via targeting ARID3A. Tissue Cell. 2022;76:101759. 10.1016/j.tice.2022.101759. 35219069 10.1016/j.tice.2022.101759

[31] Zhu K, Lin J, Chen S, Xu Q. miR-9-5p promotes lung adenocarcinoma cell proliferation, migration and invasion by targeting ID4. Technol Cancer Res Treat. 2021;20:15330338211048592. 10.1177/15330338211048592. 34723712 10.1177/15330338211048592 PMC8564129

[32] Zou P, Zhu M, Lian C, et al. miR-192-5p suppresses the progression of lung cancer bone metastasis by targeting TRIM44. Sci Rep. 2019;9(1):19619. 10.1038/s41598-019-56018-5. 31873114 10.1038/s41598-019-56018-5 PMC6928221

[33] Tang Z, Jiang Y, Ding S, Jiang S, Tang R, Luo P. miR-370 impacts the biological behavior of lung cancer cells by targeting the SMAD1 signaling pathway. Am J Transl Res. 2022;14(11):8117–28. 36505312 PMC9730076

[34] Zhang JX, Yang W, Wu JZ, et al. MicroRNA-32-5p inhibits epithelial-mesenchymal transition and metastasis in lung adenocarcinoma by targeting SMAD family 3. J Cancer. 2021;12(8):2258–67. 10.7150/jca.48387. 33758603 10.7150/jca.48387 PMC7974882

[35] Ortiz-Quintero B. Cell-free microRNAs in blood and other body fluids, as cancer biomarkers. Cell Prolif. 2016;49(3):281–303. 10.1111/cpr.12262. 27218664 10.1111/cpr.12262 PMC6496612

[36] Gayosso-Gómez LV, Ortiz-Quintero B. Circulating MicroRNAs in blood and other body fluids as biomarkers for diagnosis, prognosis, and therapy response in lung cancer. Diagnostics. 2021;11(3):421. 10.3390/diagnostics11030421. 33801442 10.3390/diagnostics11030421 PMC7999833

[37] Wang D, Hao C, Zhang L, et al. Exosomal miR-125a-5p derived from silica-exposed macrophages induces fibroblast transdifferentiation. Ecotoxicol Environ Saf. 2020;192:110253. 10.1016/j.ecoenv.2020.110253. 32059163 10.1016/j.ecoenv.2020.110253

[38] Nicoloso MS, Sun H, Spizzo R, et al. Single-nucleotide polymorphisms inside microRNA target sites influence tumor susceptibility. Cancer Res. 2010;70(7):2789–98. 10.1158/0008-5472.CAN-09-3541. 20332227 10.1158/0008-5472.CAN-09-3541 PMC2853025

[39] Li B, Chen J, Wu Y, Luo H, Ke Y. Decrease of circARID1A retards glioblastoma invasion by modulating miR-370-3p/ TGFBR2 pathway. Int J Biol Sci. 2022;18(13):5123–35. 10.7150/ijbs.66673. 35982888 10.7150/ijbs.66673 PMC9379412

[40] Wang S, Chen Y, Lei G, et al. Serum exosome-derived microRNA-193a-5p and miR-381-3p regulate adenosine 5’-Monophosphate-Activated Protein Kinase/Transforming Growth Factor Beta/Smad2/3 signaling pathway and promote fibrogenesis. Clin Transl Gastroenterol. 2024;15(2):e00662. 10.14309/ctg.000000000000066. 38099588 10.14309/ctg.000000000000066 PMC10887447

[41] Ge L, Habiel DM, Hansbro PM, et al. miR-323a-3p regulates lung fibrosis by targeting multiple profibrotic pathways. JCI Insight. 2016;1(20):e90301. 10.1172/jci.insight.90301. 27942594 10.1172/jci.insight.90301 PMC5135276

[42] Wang Y, Xue Q, Zheng Q, et al. SMAD4 mutation correlates with poor prognosis in non-small cell lung cancer. Lab Investig. 2021;101(4):463–76. 10.1038/s41374-020-00517-x. 33303972 10.1038/s41374-020-00517-x

[43] Wei J, Yu H, Liu T, Wang Z, Lang C, Pan Y. FOXA1-induced LINC00621 promotes lung adenocarcinoma progression via activating the TGF-β signaling pathway. Thorac Cancer. 2023;14(21):2026–37. 10.1111/1759-7714.14986. 37277890 10.1111/1759-7714.14986 PMC10363846

[44] Sato R, Imamura K, Semba T, et al. TGF-β signaling activated by cancer-associated fibroblasts determines the histological signature of lung adenocarcinoma. Cancer Res. 2021;81(18):canres.3941.2020. 10.1158/0008-5472.can-20-3941. 10.1158/0008-5472.can-20-3941 PMC9397619 34289987

[45] Qian C, Jiang Z, Zhou T, et al. Vesicle-mediated transport-related genes are prognostic predictors and are associated with tumor immunity in lung adenocarcinoma. Front Immunol. 2022;13:1034992. 10.3389/fimmu.2022.1034992. 36524130 10.3389/fimmu.2022.1034992 PMC9745133

[46] Cárdenas-Quesada N, Díaz-Beltrán L, Rosa-Garrido C, et al. TFG-β nuclear staining as a potential relapse risk factor in early-stage non-small-cell lung cancer. Int J Mol Sci. 2022;23(22):13780. 10.3390/ijms232213780. 36430262 10.3390/ijms232213780 PMC9694009

[47] Lee JH, Shin KM, Lee SY, et al. Genetic variant of notch regulator DTX1 predicts survival after lung cancer surgery. Ann Surg Oncol. 2019;26(11):3756–64. 10.1245/s10434-019-07614-2. 31313037 10.1245/s10434-019-07614-2

[48] Vasileva MV, Khromova NV, Kopnin BP, Dugina VB, Kopnin PB. Significance of NOTCH1 expression in the progression of human lung and colorectal cancers. Biochem (Mosc). 2022;87(10):1199–205. 10.1134/s0006297922100133. 10.1134/s0006297922100133 36273888

[49] Sebastian NT, Webb A, Shilo K, et al. A PI3K gene expression signature predicts for recurrence in early-stage non–small cell lung cancer treated with stereotactic body radiation therapy. Cancer. 2023;129(24):3971–7. 10.1002/cncr.34983. 37560930 10.1002/cncr.34983

[50] Ye Q, Falatovich B, Singh S, Ivanov AV, Eubank TD, Guo NL. A multi-omics network of a seven-gene prognostic signature for non-small cell lung cancer. Int J Mol Sci. 2021;23(1):219. 10.3390/ijms23010219. 35008645 10.3390/ijms23010219 PMC8745553

[51] Zhao J, Li X, Liu L, Zhu Z, He C. Exosomes in lung cancer metastasis, diagnosis, and immunologically relevant advances. Front Immunol. 2023;14:1326667. 10.3389/fimmu.2023.1326667. 38155975 10.3389/fimmu.2023.1326667 PMC10752943

[52] Yu F, Liang M, Huang Y, Wu W, Zheng B, Chen C. Hypoxic tumor-derived exosomal miR-31-5p promotes lung adenocarcinoma metastasis by negatively regulating SATB2-reversed EMT and activating MEK/ERK signaling. J Exp Clin Cancer Res. 2021;40(1):179. 10.1186/s13046-021-01979-7. 34074322 10.1186/s13046-021-01979-7 PMC8167983

[53] Zhang X, Sai B, Wang F, et al. Hypoxic BMSC-derived exosomal miRNAs promote metastasis of lung cancer cells via STAT3-induced EMT. Mol Cancer. 2019;18(1):40. 10.1186/s12943-019-0959-5. 30866952 10.1186/s12943-019-0959-5 PMC6417285

[54] Chang RM, Fu Y, Zeng J, Zhu XY, Gao Y. Cancer-derived exosomal miR-197-3p confers angiogenesis via targeting TIMP2/3 in lung adenocarcinoma metastasis. Cell Death Dis. 2022;13(12):1032. 10.1038/s41419-022-05420-5. 36494333 10.1038/s41419-022-05420-5 PMC9734149

[55] Hsu YL, Hung JY, Chang WA, et al. Hypoxic lung cancer-secreted exosomal miR-23a increased angiogenesis and vascular permeability by targeting prolyl hydroxylase and tight junction protein ZO-1. Oncogene. 2017;36(34):4929–42. 10.1038/onc.2017.105. 28436951 10.1038/onc.2017.105

[56] Luo C, Xin H, Zhou Z, et al. Tumor-derived exosomes induce immunosuppressive macrophages to foster intrahepatic cholangiocarcinoma progression. Hepatology. 2022;76(4):982–99. 10.1002/hep.32387. 35106794 10.1002/hep.32387

[57] Poggio M, Hu T, Pai CC, et al. Suppression of exosomal PD-L1 induces systemic anti-tumor immunity and memory. Cell. 2019;177(2):414-427.e13. 10.1016/j.cell.2019.02.016. 30951669 10.1016/j.cell.2019.02.016 PMC6499401

[58] Konoshenko MY, Lekchnov EA, Vlassov AV, Laktionov PP. Isolation of extracellular vesicles. Biomed Res Int. 2018;2018:8545347. 10.1155/2018/8545347. 29662902 10.1155/2018/8545347 PMC5831698

