# Supplementary material for: Circulating cell-free and extracellular vesicles-derived microRNA as prognostic biomarkers in patients with early-stage NSCLC: results from RESTING study
Source: J Exp Clin Cancer Res. 2024 Aug 22;43:241. doi: 10.1186/s13046-024-03156-y (PMC11340091; doi:10.1186/s13046-024-03156-y)
Supplement: Supplementary file 1 — Supplementary Material 1 [file 13046_2024_3156_MOESM1_ESM.docx]

**Supplementary Figure 1. Characterization of extracellular vesicles.**

**
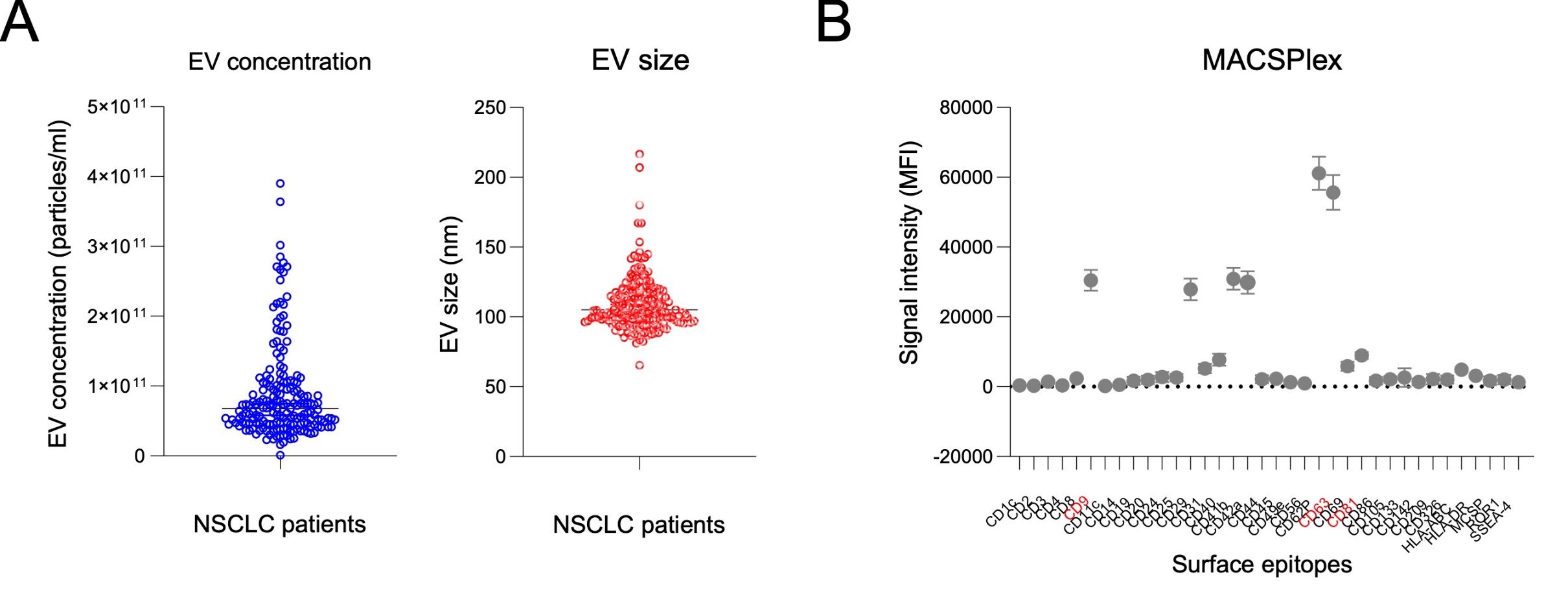
**

# **(A) Boxplot representation of average plasma concentration (particles/mL) and mean size (nm) of patients’s extracellular vesicles (EVs), as quantified at theNanoSight tracking system. (B) Average expression of EV-specific surface epitopes, indicated as Mean Fluorescence Intensity (MFI) as quantified by flow cytometry with the MACSPlex Exosome Kit (values have been normalized to blank controls). The most relevant EV markers CD9, CD63, and CD81 are highlighted in red.**

**Supplementary Figure 2. Bioinformatic analysis**

**Supplementary Figure 3.** Patient disposal for the main study analyses

222 stage I-IIIA NSCLCs

CF-miRNA EV-miRNA


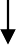


8 missing for the PFs

7 missing for the PFs

24 poor quality sample

19 poor quality sample

195 available for DFS analysis

156 avalilable for combined analysis of CF- and EV-miRNA

163 available for statistical mainstream analysis

169 available for statistical mainstream analysis

27 missing for DFS

DFS: disease-free survival; PF: prognostic factors; CF: cell-free; EV: extracellular vesicle

# **Supplementary Figure 4.** miRNAs and demographic and clinical covariates. **A)** CF-miRNAs in relation to sex, histotype and ECOG PS. **B)** EV-miRNAs in relation to sex and pathological stage.


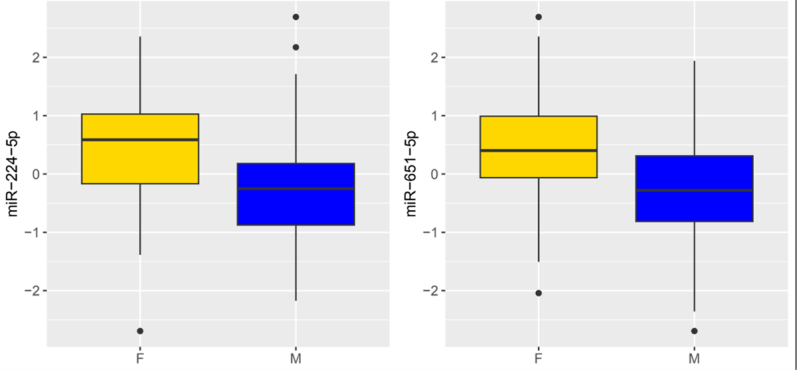

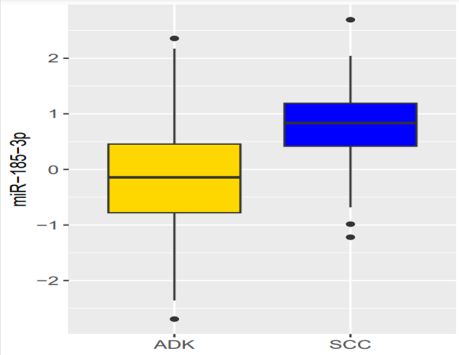

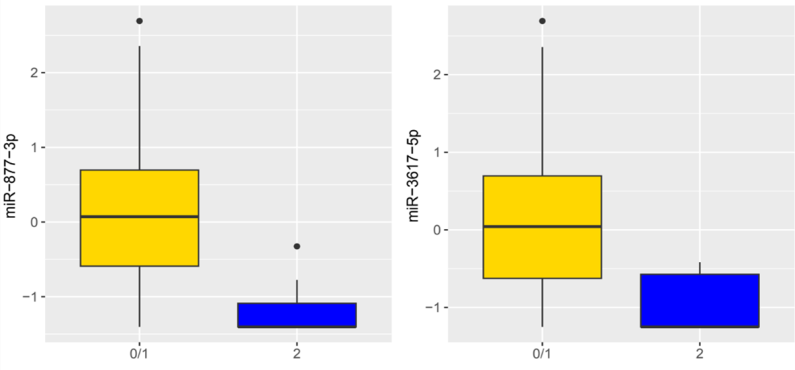


**A**

**Histotype**

**Sex**

**ECOG PS**


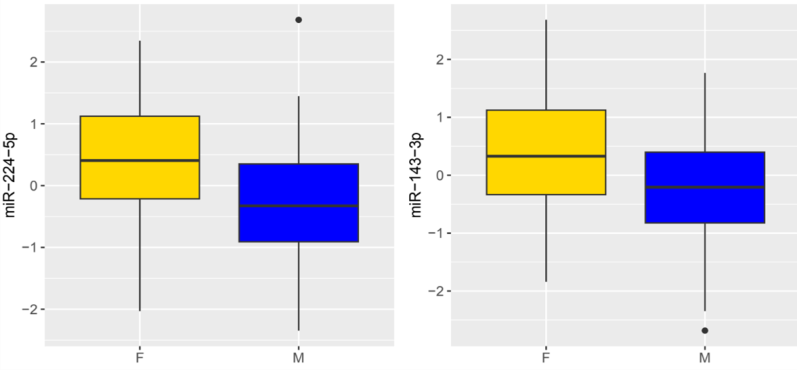

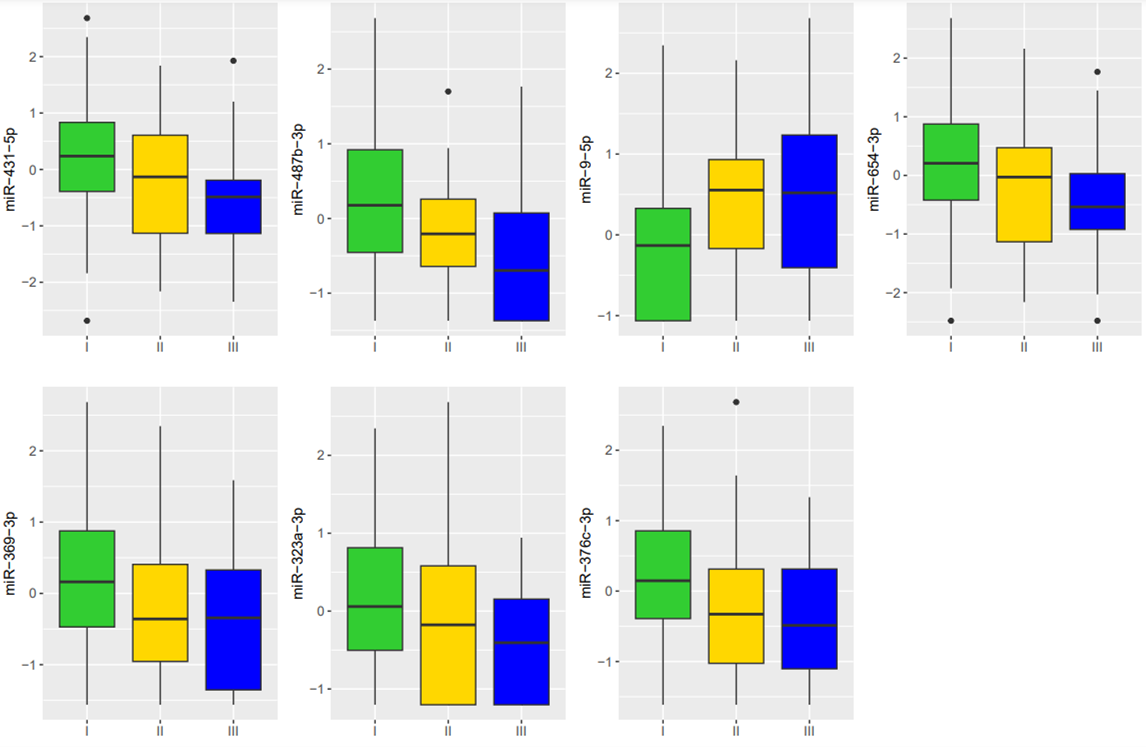


**B**

**Sex**

**Stage**

CF: Cell-Free; ECOG: Eastern Cooperative Oncology Group; PS: Performance status; EV: extracellular vesicle

**Supplementary Figure 5.** Kaplan-Meier curve for disease-free survival (DFS) by pathologic stage


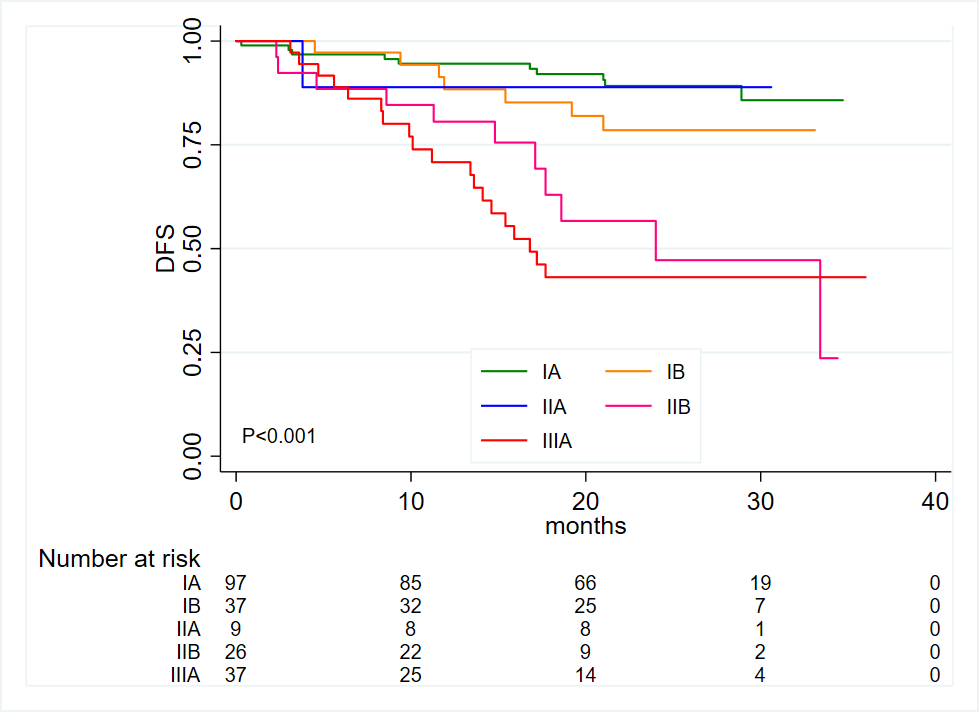


**Supplementary Figure 6.** Performance of the models using cell-free and extracellular vesicle miRNA data

A B C


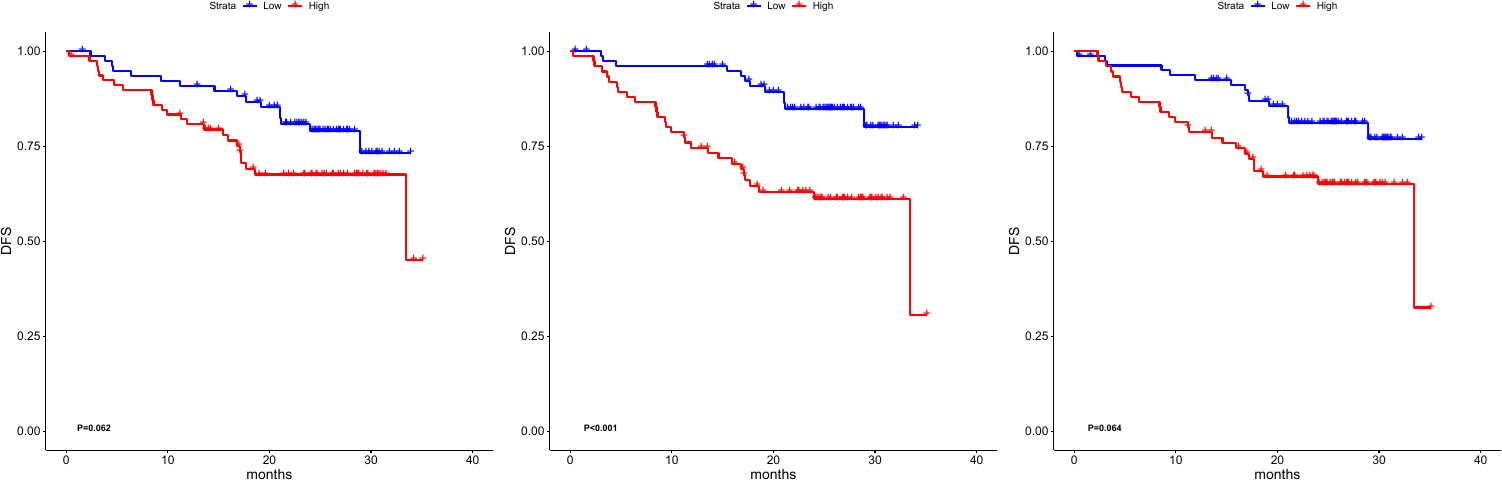


D E F


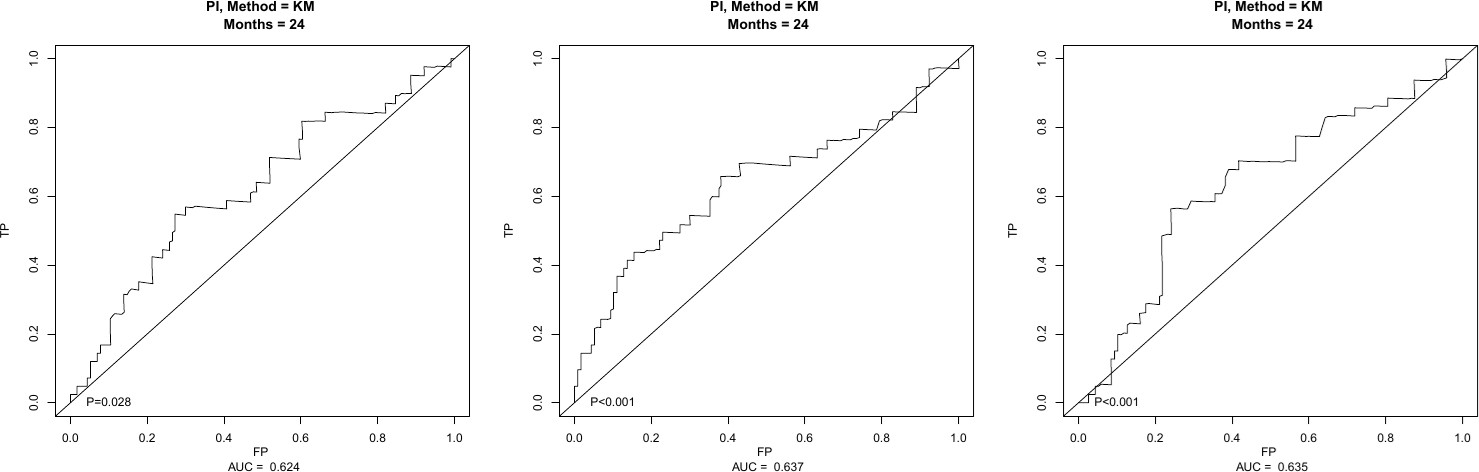


# From (A) to (C) 5-fold cross-validated Kaplan-Meier curves for the models derived using the miRNA data alone, miRNA and basic prognostic factors (age, sex, and pathologic stage) data, and prognostic factors data alone. From (D) to (F) Five- fold cross-validated time-dependent ROC curves (at 24 months) for the three models. DFS: disease-free survival.

**Supplementary Table 1**. Results from univariable Cox regression models

|  | **HR** | **(95% CI)** | **p-value** |
| --- | --- | --- | --- |
| **Sex** |  |  |  |
| F | 1.00 |  | **0.027** |
| M | 2.03 | 1.08 – 3.81 |  |
| **Age at surgery (yrs) - continuous** | 1.03 | 0.99 – 1.07 | 0.157 |
| **Age at surgery (yrs)** |  |  |  |
| < 70 | 1.00 |  |  |
| ≥ 70 | 1.70 | 0.96 – 2.99 | **0.066** |
| **Smoking habit** |  |  |  |
| Never smoker | 1.00 |  |  |
| Ex-smoker | 1.45 | 0.56 – 3.73 | 0.445 |
| Current smoker | 1.53 | 0.54 – 4.30 | 0.421 |
| **ECOG PS** |  |  |  |
| 0/1 | 1.00 |  |  |
| 2 | 6.04 | 2.67 – 13.64 | **<0.001** |
| **Type of resection** |  |  |  |
| Atypical resection | 1.00 |  |  |
| Pneumectomy | 2.57 | 0.68 – 8.65 | 0.163 |
| Lobectomy | 0.92 | 0.36 – 2.32 | 0.856 |
| **Radicality** |  |  |  |
| R0 | 1.00 |  |  |
| R1/R2 | 2.15 | 0.52 – 8.87 | 0.288 |
| **Histological diagnosis** |  |  |  |
| Adenocarcinoma | 1.00 |  |  |
| Squamous cell carcinoma | 1.54 | 0.80 – 2.93 | 0.194 |
| **Pathological stage** |  |  |  |
| I | 1.00 |  |  |
| II | 3.05 | 1.45 – 6.41 | **0.003** |
| IIIA | 5.31 | 2.75 – 10.25 | **<0.001** |
| **Grading** |  |  |  |
| G1 | 1.00 |  |  |
| G2 | 2.41 | 0.73 – 7.95 | 0.149 |
| G3 | 2.01 | 0.55 – 7.45 | 0.294 |

HR: hazard ratio; CI: confidence intervals; ECOG PS: Eastern Cooperative Oncology Group Performance Status.

**Supplementary Table 2**. Multivariable Cox proportional hazards regression models on disease-free survival (analyses considering together CF- and EV-miRNA)

|  | **Model 1** | **Model 2** | **Model 3** | | |
| --- | --- | --- | --- | --- | --- |
|  | **Coef.** | **Coef.** | **Coef.** | **HR (95% CI)** | **p-value** |
| CF-miR-135a-5p | -0.190 | -0.052 |  |  |  |
| CF-miR-29c-3p | 0.186 | 0.086 |  |  |  |
| CF-miR-877-3p | -0.423 | -0.224 |  |  |  |
| EV-miR-192-5p | 0.111 | 0.066 |  |  |  |
| CF-miR-1468-5p | 0.069 |  |  |  |  |
| CF-miR-3064-5p | -0.029 |  |  |  |  |
| CF-miR-3613-3p | 0.034 |  |  |  |  |
| CF-miR-3913-5p | -0.102 |  |  |  |  |
| EV-miR-127-3p | -0.105 |  |  |  |  |
| EV-miR-1277-3p | -0.083 |  |  |  |  |
| EV-miR-181a-2-3p | -0.045 |  |  |  |  |
| EV-miR-329-3p | -0.017 |  |  |  |  |
| EV-miR-5187-5p | 0.089 |  |  |  |  |
| EV-miR-532-3p | -0.052 |  |  |  |  |
| Sex (M vs F) |  | 0.137 | 0.166 | 1.18 (0.61-2.30) | 0.626 |
| Age at surgery |  | 0.014 | 0.015 | 1.02 (0.97-1.06) | 0.508 |
| pSTAGE (II vs I) |  | 0.997 | 1.008 | 2.74 (1.26-5.94) | 0.011 |
| pSTAGE (IIIA vs I) |  | 1.297 | 1.404 | 4.07 (1.97-3.79) | <0.001 |

CF: cell-free; EV: extracellular vesicle; HR: hazard ratio; CI: confidence intervals; pSTAGE: pathologic disease stage; Coef: beta regression coefficient.

Model 1 refers to the results obtained fitting an elastic net penalized Cox model on the microRNA data only; Model 2 refers to the results obtained fitting an elastic net penalized Cox model on the microRNA and basic prognostic factors data; Model 3 refers to the results obtained fitting a standard Cox model only on the data of basic prognostic factors.

# **Supplementary Table 3**. List of miRNAs whose expression was correlated with at least one of the 24 miRNAs identified by the mainstream statistical analyses (Model 1)

| **List of miRNA from Model 1** | **Pearson correlation coefficient** | **List of correlated miRNAs** |
| --- | --- | --- |
| miR-135a-5p | - | - |
| miR-877-3p | - | - |
| miR-29c-3p | 0.8384 | miR-30e-5p |
|  | 0.7726 | miR-15a-5p |
|  | 0.7644 | miR-29a-3p |
|  | 0.7378 | miR-101-3p |
|  | 0.7139 | miR-144-5p |
|  | 0.6791 | miR-140-3p |
|  | -0.6489 | miR-98-5p |
|  | 0.6375 | miR-144-3p |
|  | 0.6122 | miR-96-5p |
|  | 0.6115 | miR-660-5p |
|  | 0.6072 | miR-194-5p |
|  | 0.6064 | miR-15b-5p |
|  | 0.6063 | miR-30d-5p |
| miR-182-5p | 0.8766 | miR-183-5p |
|  | 0.6549 | miR-486-5p |
|  | 0.6466 | miR-190a-5p |
|  | 0.6079 | miR-4732-5p |
| miR-192-5p | 0.6492 | miR-194-5p |
|  | 0.6099 | miR-144-3p |
| miR-532-3p | - | - |
| miR-589-5p | - | - |
| miR-127-3p | 0.6848 | miR-431-5p |
|  | 0.653 | miR-493-5p |
|  | 0.6346 | miR-654-3p |
|  | 0.6259 | miR-411-5p |
|  | 0.6019 | miR-379-5p |
| miR-181a-2-3p | - | - |
| miR-1277-3p | - | - |
| miR-136-3p | 0.7044 | miR-376c-3p |
|  | 0.6025 | miR-381-3p |
| miR-18a-5p | - | - |
| miR-32-5p | 0.6808 | miR-451a |
|  | 0.6294 | miR-30e-5p |
|  | -0.6172 | miR-744-5p |
|  | 0.6052 | miR-15a-5p |
| miR-323b-3p | 0.6849 | miR-654-3p |
|  | 0.6636 | miR-431-5p |

|  | 0.6498 | miR-485-3p |
| --- | --- | --- |
|  | 0.6496 | miR-487b-3p |
|  | 0.6473 | miR-409-3p |
|  | 0.6374 | miR-323a-3p |
|  | 0.618 | miR-369-5p |
|  | 0.6002 | miR-370-3p |
| miR-328-3p | 0.6248 | miR-223-3p |
| miR-339-3p | - | - |
| miR-361-3p | - | - |
| miR-3615 | - | - |
| miR-370-3p | 0.7601 | miR-431-5p |
|  | 0.7079 | miR-409-3p |
|  | 0.6808 | miR-654-3p |
|  | 0.6775 | miR-379-5p |
|  | 0.6762 | miR-134-5p |
|  | 0.6589 | miR-493-5p |
|  | 0.6582 | miR-382-5p |
|  | 0.6571 | miR-369-5p |
|  | 0.6381 | miR-432-5p |
|  | 0.6002 | miR-323b-3p |
| miR-5187-5p | - | - |
| miR-628-5p | - | - |
| miR-6852-5p | - | - |
| miR-9-5p | - | - |
| miR-99b-5p | 0.8816 | miR-125a-5p |

**Supplementary Table 4.** Results of pathway enrichment analyzing the 3 CF-miRNAs associated with disease- free survival, together with their correlated miRNAs

| **Pathway** | **Total** | **Expected** | **Hits** | **p-value** | **FDR** | **RichScore** |
| --- | --- | --- | --- | --- | --- | --- |
| Inflammasomes | 17 | 0,0403 | 2 | 0.0007 | 0,0316 | 11,76 |
| Antigen Presentation: Folding assembly  and peptide loading of class I MHC | 24 | 0,0569 | 2 | 0.0014 | 0,0316 | 8,33 |
| Cyclin D associated events in G1 | 30 | 0,0711 | 2 | 0.0022 | 0,0316 | 6,67 |
| G1 Phase | 30 | 0,0711 | 2 | 0.0022 | 0,0316 | 6,67 |
| Nucleotide-binding domain leucine rich repeat containing receptor (NLR)  signaling pathways | 44 | 0,104 | 2 | 0.0047 | 0,0473 | 4,55 |
| MHC class II antigen presentation | 89 | 0,211 | 3 | 0.0011 | 0,0316 | 3,37 |
| Mitotic G1-G1/S phases | 126 | 0,299 | 4 | 0.0002 | 0,0177 | 3,17 |
| Membrane Trafficking | 146 | 0,346 | 3 | 0.0045 | 0,0473 | 2,05 |
| Cell Cycle Mitotic | 409 | 0,97 | 5 | 0.002 | 0,0316 | 1,22 |
| Cell Cycle | 498 | 1,18 | 5 | 0.0047 | 0,0473 | 1 |

FDR: False Discovery Rate

**Supplementary Table 5**. Results of pathway enrichment analyzing the 21 EV-miRNAs associated with disease-free survival, together with their correlated miRNAs

|  |  |  |  |  |  |  |
| --- | --- | --- | --- | --- | --- | --- |
| **Pathway** | **Total** | **Expected** | **Hits** | **p-value** | **FDR** | **RichScore** |
| Loss of Function of SMAD2/3 in Cancer | 7 | 0,15 | 3 | 0,0003 | 0,0006 | 42,9 |
| Loss of Function of TGFBR1 in Cancer | 7 | 0,15 | 3 | 0,0003 | 0,0006 | 42,9 |
| Post-transcriptional silencing by small  RNAs | 7 | 0,15 | 3 | 0,0003 | 0,0006 | 42,9 |
| Signaling by TGF-beta Receptor Complex in Cancer | 8 | 0,172 | 3 | 0,0005 | 0,0008 | 37,5 |
| AKT phosphorylates targets in the cytosol | 11 | 0,236 | 4 | 6,00E-05 | 0,0001 | 36,4 |
| Signaling by Activin | 12 | 0,258 | 4 | 8,85E-05 | 0,0002 | 33,3 |
| ER to Golgi Transport | 9 | 0,193 | 3 | 0,0007 | 0,001 | 33,3 |
| COPII (Coat Protein 2) Mediated Vesicle Transport | 9 | 0,193 | 3 | 0,0007 | 0,001 | 33,3 |
| Pre-NOTCH Transcription and Translation | 19 | 0,408 | 6 | 1,91E-06 | 5,30E-06 | 31,6 |
| E2F-enabled inhibition of pre-replication complex formation | 10 | 0,215 | 3 | 0,001 | 0,0013 | 30,0 |
| Activation of the AP-1 family of transcription factors | 10 | 0,215 | 3 | 0,001 | 0,0013 | 30,0 |
| Constitutive Signaling by AKT1 E17K in  Cancer | 22 | 0,473 | 6 | 4,98E-06 | 1,24E-05 | 27,3 |
| Pre-NOTCH Expression and Processing | 24 | 0,516 | 6 | 8,67E-06 | 2,06E-05 | 25,0 |
| Chk1/Chk2(Cds1) mediated inactivation of  Cyclin B:Cdk1 complex | 12 | 0,258 | 3 | 0,0019 | 0,0019 | 25,0 |
| SHC-related events triggered by IGF1R | 18 | 0,387 | 4 | 0,0005 | 0,0008 | 22,2 |
| Signaling by NODAL | 19 | 0,408 | 4 | 0,0006 | 0,0009 | 21,1 |
| Signaling by FGFR3 mutants | 22 | 0,473 | 4 | 0,0011 | 0,0013 | 18,2 |
| Signaling by BMP | 23 | 0,494 | 4 | 0,0013 | 0,0015 | 17,4 |
| Oncogene Induced Senescence | 30 | 0,645 | 5 | 0,0004 | 0,0007 | 16,7 |
| SHC1 events in ERBB2 signaling | 24 | 0,516 | 4 | 0,0016 | 0,0016 | 16,7 |
| Antigen Presentation: Folding | 24 | 0,516 | 4 | 0,0016 | 0,0016 | 16,7 |
| FCERI mediated MAPK activation | 37 | 0,795 | 6 | 0,0001 | 0,0003 | 16,2 |
| Downregulation of TGF-beta receptor  signaling | 26 | 0,559 | 4 | 0,0021 | 0,0021 | 15,4 |
| PI3K events in ERBB4 signaling | 94 | 2,02 | 14 | 9,50E-09 | 1,06E-07 | 14,9 |
| PIP3 activates AKT signaling | 94 | 2,02 | 14 | 9,50E-09 | 1,06E-07 | 14,9 |
| PI3K events in ERBB2 signaling | 94 | 2,02 | 14 | 9,50E-09 | 1,06E-07 | 14,9 |
| PI-3K cascade:FGFR1-4 | 94 | 2,02 | 14 | 9,50E-09 | 1,06E-07 | 14,9 |
| PI3K/AKT activation | 97 | 2,08 | 14 | 1,44E-08 | 1,38E-07 | 14,4 |
| GAB1 signalosome | 98 | 2,11 | 14 | 1,65E-08 | 1,38E-07 | 14,3 |
| Role of LAT2/NTAL/LAB on calcium  mobilization | 103 | 2,21 | 14 | 3,18E-08 | 1,87E-07 | 13,6 |
| Signaling by SCF-KIT | 133 | 2,86 | 18 | 3,20E-10 | 1,81E-08 | 13,5 |
| Signalling to ERKs | 37 | 0,795 | 5 | 0,0011 | 0,0013 | 13,5 |

| Regulation of cholesterol biosynthesis by  SREBP (SREBF) | 39 | 0,838 | 5 | 0,0014 | 0,0015 | 12,8 |
| --- | --- | --- | --- | --- | --- | --- |
| Oxidative Stress Induced Senescence | 88 | 1,89 | 11 | 2,39E-06 | 6,29E-06 | 12,5 |
| TRAF6 Mediated Induction of  proinflammatory cytokines | 56 | 1,2 | 7 | 0,0002 | 0,0004 | 12,5 |
| PI3K/AKT Signaling in Cancer | 81 | 1,74 | 10 | 7,83E-06 | 1,91E-05 | 12,3 |
| Fc epsilon receptor (FCERI) signaling | 169 | 3,63 | 20 | 3,62E-10 | 1,81E-08 | 11,8 |
| Downstream signaling of activated FGFR1 | 139 | 2,99 | 16 | 3,55E-08 | 1,87E-07 | 11,5 |
| Downstream signaling of activated FGFR2 | 139 | 2,99 | 16 | 3,55E-08 | 1,87E-07 | 11,5 |
| Downstream signaling of activated FGFR3 | 139 | 2,99 | 16 | 3,55E-08 | 1,87E-07 | 11,5 |
| Downstream signaling of activated FGFR4 | 139 | 2,99 | 16 | 3,55E-08 | 1,87E-07 | 11,5 |
| Downstream signal transduction | 151 | 3,24 | 17 | 1,80E-08 | 1,38E-07 | 11,3 |
| Signaling by ERBB4 | 143 | 3,07 | 16 | 5,33E-08 | 2,66E-07 | 11,2 |
| Unfolded Protein Response (UPR) | 73 | 1,57 | 8 | 0,0002 | 0,0003 | 11,0 |
| TCR signaling | 64 | 1,38 | 7 | 0,0004 | 0,0007 | 10,9 |
| Signaling by FGFR | 151 | 3,24 | 16 | 1,15E-07 | 4,26E-07 | 10,6 |
| Signaling by FGFR1 | 151 | 3,24 | 16 | 1,15E-07 | 4,26E-07 | 10,6 |
| Signaling by FGFR2 | 151 | 3,24 | 16 | 1,15E-07 | 4,26E-07 | 10,6 |
| Signaling by FGFR3 | 151 | 3,24 | 16 | 1,15E-07 | 4,26E-07 | 10,6 |
| Signaling by FGFR4 | 151 | 3,24 | 16 | 1,15E-07 | 4,26E-07 | 10,6 |
| Signaling by ERBB2 | 152 | 3,27 | 16 | 1,27E-07 | 4,54E-07 | 10,5 |
| Cellular Senescence | 143 | 3,07 | 15 | 3,37E-07 | 1,09E-06 | 10,5 |
| Toll Like Receptor 10 (TLR10) Cascade | 67 | 1,44 | 7 | 0,0005 | 0,0008 | 10,4 |
| Toll Like Receptor 5 (TLR5) Cascade | 67 | 1,44 | 7 | 0,0005 | 0,0008 | 10,4 |
| MyD88 cascade initiated on plasma  membrane | 67 | 1,44 | 7 | 0,0005 | 0,0008 | 10,4 |
| DAP12 signaling | 154 | 3,31 | 16 | 1,52E-07 | 5,24E-07 | 10,4 |
| Downstream signaling events of B Cell  Receptor (BCR) | 164 | 3,52 | 17 | 6,25E-08 | 2,98E-07 | 10,4 |
| IRE1alpha activates chaperones | 58 | 1,25 | 6 | 0,0014 | 0,0015 | 10,3 |
| TRAF6 mediated induction of NFkB and  MAP kinases upon TLR7/8 or 9 activation | 68 | 1,46 | 7 | 0,0006 | 0,0009 | 10,3 |
| Signaling by PDGF | 177 | 3,8 | 18 | 3,35E-08 | 1,87E-07 | 10,2 |
| Toll Like Receptor 7/8 (TLR7/8) Cascade | 69 | 1,48 | 7 | 0,0006 | 0,0009 | 10,1 |
| MyD88 dependent cascade initiated on  endosome | 69 | 1,48 | 7 | 0,0006 | 0,0009 | 10,1 |
| Signaling by EGFR | 168 | 3,61 | 17 | 8,94E-08 | 4,06E-07 | 10,1 |
| NGF signalling via TRKA from the plasma  membrane | 189 | 4,06 | 19 | 1,63E-08 | 1,38E-07 | 10,1 |
| Toll Like Receptor 9 (TLR9) Cascade | 71 | 1,53 | 7 | 0,0008 | 0,001 | 9,9 |
| VEGFA-VEGFR2 Pathway | 92 | 1,98 | 9 | 0,0001 | 0,0003 | 9,8 |
| Signaling by TGF-beta Receptor Complex | 72 | 1,55 | 7 | 0,0008 | 0,0011 | 9,7 |
| IRS-related events triggered by IGF1R | 84 | 1,8 | 8 | 0,0004 | 0,0007 | 9,5 |
| MyD88:Mal cascade initiated on plasma  membrane | 74 | 1,59 | 7 | 0,001 | 0,0012 | 9,5 |
| Toll Like Receptor TLR1:TLR2 Cascade | 74 | 1,59 | 7 | 0,001 | 0,0012 | 9,5 |

| Toll Like Receptor TLR6:TLR2 Cascade | 74 | 1,59 | 7 | 0,001 | 0,0012 | 9,5 |
| --- | --- | --- | --- | --- | --- | --- |
| Toll Like Receptor 2 (TLR2) Cascade | 74 | 1,59 | 7 | 0,001 | 0,0012 | 9,5 |
| DAP12 interactions | 171 | 3,67 | 16 | 6,46E-07 | 1,90E-06 | 9,4 |
| Signaling by Type 1 Insulin-like Growth  Factor 1 Receptor (IGF1R) | 87 | 1,87 | 8 | 0,0005 | 0,0008 | 9,2 |
| IGF1R signaling cascade | 87 | 1,87 | 8 | 0,0005 | 0,0008 | 9,2 |
| Insulin receptor signalling cascade | 87 | 1,87 | 8 | 0,0005 | 0,0008 | 9,2 |
| TRIF-mediated TLR3/TLR4 signaling | 77 | 1,65 | 7 | 0,0013 | 0,0014 | 9,1 |
| Signaling by VEGF | 100 | 2,15 | 9 | 0,0003 | 0,0005 | 9,0 |
| MyD88-independent TLR3/TLR4 cascade | 78 | 1,68 | 7 | 0,0014 | 0,0015 | 9,0 |
| Toll Like Receptor 3 (TLR3) Cascade | 78 | 1,68 | 7 | 0,0014 | 0,0015 | 9,0 |
| Signaling by the B Cell Receptor (BCR) | 190 | 4,08 | 17 | 5,35E-07 | 1,67E-06 | 8,9 |
| IRS-mediated signalling | 80 | 1,72 | 7 | 0,0016 | 0,0016 | 8,8 |
| IRS-related events | 82 | 1,76 | 7 | 0,0018 | 0,0019 | 8,5 |
| Signaling by Insulin receptor | 111 | 2,38 | 9 | 0,0006 | 0,0009 | 8,1 |
| Signalling by NGF | 273 | 5,87 | 21 | 2,94E-07 | 9,80E-07 | 7,7 |
| Signaling by Interleukins | 107 | 2,3 | 8 | 0,002 | 0,0021 | 7,5 |
| Cellular responses to stress | 256 | 5,5 | 19 | 1,96E-06 | 5,30E-06 | 7,4 |
| Cell Cycle Checkpoints | 123 | 2,64 | 9 | 0,0013 | 0,0014 | 7,3 |
| Generic Transcription Pathway | 189 | 4,06 | 13 | 0,0002 | 0,0004 | 6,9 |
| Diseases of signal transduction | 235 | 5,05 | 15 | 0,0001 | 0,0003 | 6,4 |
| Adaptive Immune System | 430 | 9,24 | 26 | 1,15E-06 | 3,29E-06 | 6,0 |
| Innate Immune System | 569 | 12,2 | 30 | 2,74E-06 | 7,03E-06 | 5,3 |
| Developmental Biology | 438 | 9,41 | 22 | 0,0001 | 0,0003 | 5,0 |
| Immune System | 942 | 20,2 | 43 | 5,87E-07 | 1,78E-06 | 4,6 |

FDR: False Discovery Rate

**Supplementary Table 6.** Results of pathway enrichment analyzing the EV-miRNAs highly expressed in patients with worse outcome, together with their correlated miRNAs

|  |  |  |  |  |  |  |
| --- | --- | --- | --- | --- | --- | --- |
| **Pathway** | **Total** | **Expected** | **Hits** | **p-value** | **FDR** | **RichScore** |
| ER to Golgi Transport | 9 | 0,0507 | 2 | 0.0011 | 0,0164 | 22.2222 |
| COPII (Coat Protein 2) Mediated Vesicle  Transport | 9 | 0,0507 | 2 | 0.0011 | 0,0164 | 22.2222 |
| E2F-enabled inhibition of pre-replication  complex formation | 10 | 0,0563 | 2 | 0.0014 | 0,0164 | 20 |
| Regulation of signaling by NODAL | 10 | 0,0563 | 2 | 0.0014 | 0,0164 | 20 |
| Signaling by Activin | 12 | 0,0676 | 2 | 0.002 | 0,0197 | 16.6667 |
| Phosphorylation of Emi1 | 6 | 0,0338 | 1 | 0.0333 | 0,0422 | 16.6667 |
| FGFR1b ligand binding and activation | 6 | 0,0338 | 1 | 0.0333 | 0,0422 | 16.6667 |
| MASTL Facilitates Mitotic Progression | 6 | 0,0338 | 1 | 0.0333 | 0,0422 | 16.6667 |
| Activation of NIMA Kinases NEK9,6,7 | 6 | 0,0338 | 1 | 0.0333 | 0,0422 | 16.6667 |
| Assembly of the ORC complex at the origin  of replication | 6 | 0,0338 | 1 | 0.0333 | 0,0422 | 16.6667 |
| Nef Mediated CD8 Down-regulation | 7 | 0,0394 | 1 | 0.0388 | 0,0462 | 14.2857 |
| Vitamin C (ascorbate) metabolism | 7 | 0,0394 | 1 | 0.0388 | 0,0462 | 14.2857 |
| Post-transcriptional silencing by small  RNAs | 7 | 0,0394 | 1 | 0.0388 | 0,0462 | 14.2857 |
| Transport of nucleosides and free purine and pyrimidine bases across the plasma  membrane | 7 | 0,0394 | 1 | 0.0388 | 0,0462 | 14.2857 |
| Antigen Presentation: Folding assembly  and peptide loading of class I MHC | 24 | 0,135 | 3 | 0.0003 | 0,0164 | 12.5 |
| BH3-only proteins associate with and  inactivate anti-apoptotic BCL-2 members | 8 | 0,045 | 1 | 0.0442 | 0,0491 | 12.5 |
| ATF6-alpha activates chaperones | 8 | 0,045 | 1 | 0.0442 | 0,0491 | 12.5 |
| Formyl peptide receptors bind formyl  peptides and many other ligands | 8 | 0,045 | 1 | 0.0442 | 0,0491 | 12.5 |
| CDC6 association with the ORC:origin  complex | 8 | 0,045 | 1 | 0.0442 | 0,0491 | 12.5 |
| Inflammasomes | 17 | 0,0957 | 2 | 0.004 | 0,0234 | 11.7647 |
| Signaling by NODAL | 19 | 0,107 | 2 | 0.005 | 0,0254 | 10.5263 |
| Pre-NOTCH Transcription and Translation | 19 | 0,107 | 2 | 0.005 | 0,0254 | 10.5263 |
| Signaling by BMP | 23 | 0,13 | 2 | 0.0073 | 0,0259 | 8.6957 |
| Pre-NOTCH Expression and Processing | 24 | 0,135 | 2 | 0.0079 | 0,0259 | 8.3333 |
| Lysosome Vesicle Biogenesis | 25 | 0,141 | 2 | 0.0085 | 0,0259 | 8 |
| FRS-mediated FGFR3 signaling | 27 | 0,152 | 2 | 0.0099 | 0,0259 | 7.4074 |
| FRS2-mediated FGFR4 signaling | 29 | 0,163 | 2 | 0.0114 | 0,0271 | 6.8966 |
| E2F mediated regulation of DNA  replication | 30 | 0,169 | 2 | 0.0122 | 0,0271 | 6.6667 |
| FRS-mediated FGFR1 signaling | 30 | 0,169 | 2 | 0.0122 | 0,0271 | 6.6667 |
| Cyclin D associated events in G1 | 30 | 0,169 | 2 | 0.0122 | 0,0271 | 6.6667 |
| G1 Phase | 30 | 0,169 | 2 | 0.0122 | 0,0271 | 6.6667 |

| FRS-mediated FGFR2 signaling | 32 | 0,18 | 2 | 0.0138 | 0,0282 | 6.25 |
| --- | --- | --- | --- | --- | --- | --- |
| Transport to the Golgi and subsequent  modification | 36 | 0,203 | 2 | 0.0172 | 0,0293 | 5.5556 |
| FCERI mediated MAPK activation | 37 | 0,208 | 2 | 0.0182 | 0,0303 | 5.4054 |
| Regulation of cholesterol biosynthesis by  SREBP (SREBF) | 39 | 0,22 | 2 | 0.0201 | 0,0319 | 5.1282 |
| Netrin-1 signaling | 42 | 0,236 | 2 | 0.0231 | 0,036 | 4.7619 |
| trans-Golgi Network Vesicle Budding | 43 | 0,242 | 2 | 0.0241 | 0,036 | 4.6512 |
| Clathrin derived vesicle budding | 43 | 0,242 | 2 | 0.0241 | 0,036 | 4.6512 |
| Nucleotide-binding domain leucine rich repeat containing receptor (NLR) signaling  pathways | 44 | 0,248 | 2 | 0.0252 | 0,0367 | 4.5455 |
| MHC class II antigen presentation | 89 | 0,501 | 4 | 0.0015 | 0,0164 | 4.4944 |
| MAP kinase activation in TLR cascade | 49 | 0,276 | 2 | 0.0307 | 0,0422 | 4.0816 |
| G2/M Checkpoints | 51 | 0,287 | 2 | 0.0331 | 0,0422 | 3.9216 |
| Regulation of actin dynamics for  phagocytic cup formation | 52 | 0,293 | 2 | 0.0343 | 0,0429 | 3.8462 |
| TRAF6 Mediated Induction of  proinflammatory cytokines | 56 | 0,315 | 2 | 0.0393 | 0,0462 | 3.5714 |
| IRE1alpha activates chaperones | 58 | 0,327 | 2 | 0.0419 | 0,0487 | 3.4483 |
| Membrane Trafficking | 146 | 0,822 | 5 | 0.0013 | 0,0164 | 3.4247 |
| Class I MHC mediated antigen processing  & presentation | 93 | 0,524 | 3 | 0.0151 | 0,0282 | 3.2258 |
| PI3K events in ERBB4 signaling | 94 | 0,529 | 3 | 0.0155 | 0,0282 | 3.1915 |
| PIP3 activates AKT signaling | 94 | 0,529 | 3 | 0.0155 | 0,0282 | 3.1915 |
| PI3K events in ERBB2 signaling | 94 | 0,529 | 3 | 0.0155 | 0,0282 | 3.1915 |
| PI-3K cascade:FGFR1 | 94 | 0,529 | 3 | 0.0155 | 0,0282 | 3.1915 |
| PI-3K cascade:FGFR2 | 94 | 0,529 | 3 | 0.0155 | 0,0282 | 3.1915 |
| PI-3K cascade:FGFR3 | 94 | 0,529 | 3 | 0.0155 | 0,0282 | 3.1915 |
| PI-3K cascade:FGFR4 | 94 | 0,529 | 3 | 0.0155 | 0,0282 | 3.1915 |
| Mitotic G1-G1/S phases | 126 | 0,709 | 4 | 0.0052 | 0,0254 | 3.1746 |
| PI3K/AKT activation | 97 | 0,546 | 3 | 0.0169 | 0,0293 | 3.0928 |
| GAB1 signalosome | 98 | 0,552 | 3 | 0.0173 | 0,0293 | 3.0612 |
| Signaling by SCF-KIT | 133 | 0,749 | 4 | 0.0063 | 0,0259 | 3.0075 |
| Signaling by EGFR | 168 | 0,946 | 5 | 0.0023 | 0,0199 | 2.9762 |
| Fc epsilon receptor (FCERI) signaling | 169 | 0,952 | 5 | 0.0024 | 0,0199 | 2.9586 |
| Role of LAT2/NTAL/LAB on calcium  mobilization | 103 | 0,58 | 3 | 0.0198 | 0,0319 | 2.9126 |
| G1/S Transition | 103 | 0,58 | 3 | 0.0198 | 0,0319 | 2.9126 |
| Downstream signaling of activated FGFR1 | 139 | 0,783 | 4 | 0.0074 | 0,0259 | 2.8777 |
| Downstream signaling of activated FGFR2 | 139 | 0,783 | 4 | 0.0074 | 0,0259 | 2.8777 |
| Downstream signaling of activated FGFR3 | 139 | 0,783 | 4 | 0.0074 | 0,0259 | 2.8777 |
| Downstream signaling of activated FGFR4 | 139 | 0,783 | 4 | 0.0074 | 0,0259 | 2.8777 |
| Signaling by ERBB4 | 143 | 0,805 | 4 | 0.0082 | 0,0259 | 2.7972 |
| Vesicle-mediated transport | 184 | 1,04 | 5 | 0.0035 | 0,0234 | 2.7174 |
| Signaling by Insulin receptor | 111 | 0,625 | 3 | 0.0241 | 0,036 | 2.7027 |

| Downstream signal transduction | 151 | 0,85 | 4 | 0.0098 | 0,0259 | 2.649 |
| --- | --- | --- | --- | --- | --- | --- |
| Signaling by FGFR | 151 | 0,85 | 4 | 0.0098 | 0,0259 | 2.649 |
| Signaling by FGFR1 | 151 | 0,85 | 4 | 0.0098 | 0,0259 | 2.649 |
| Signaling by FGFR2 | 151 | 0,85 | 4 | 0.0098 | 0,0259 | 2.649 |
| Signaling by FGFR3 | 151 | 0,85 | 4 | 0.0098 | 0,0259 | 2.649 |
| Signaling by FGFR4 | 151 | 0,85 | 4 | 0.0098 | 0,0259 | 2.649 |
| NGF signalling via TRKA from the plasma  membrane | 189 | 1,06 | 5 | 0.0039 | 0,0234 | 2.6455 |
| Signaling by ERBB2 | 152 | 0,856 | 4 | 0.0101 | 0,0259 | 2.6316 |
| DAP12 signaling | 154 | 0,867 | 4 | 0.0105 | 0,0262 | 2.5974 |
| Asparagine N-linked glycosylation | 117 | 0,659 | 3 | 0.0276 | 0,0389 | 2.5641 |
| S Phase | 117 | 0,659 | 3 | 0.0276 | 0,0389 | 2.5641 |
| Cell Cycle Checkpoints | 123 | 0,693 | 3 | 0.0314 | 0,0422 | 2.439 |
| DAP12 interactions | 171 | 0,963 | 4 | 0.015 | 0,0282 | 2.3392 |
| Signaling by PDGF | 177 | 0,997 | 4 | 0.0168 | 0,0293 | 2.2599 |
| Signalling by NGF | 273 | 1,54 | 6 | 0.0038 | 0,0234 | 2.1978 |
| Developmental Biology | 438 | 2,47 | 9 | 0.0006 | 0,0164 | 2.0548 |
| Axon guidance | 292 | 1,64 | 6 | 0.0053 | 0,0254 | 2.0548 |
| Adaptive Immune System | 430 | 2,42 | 7 | 0.0091 | 0,0259 | 1.6279 |
| Innate Immune System | 569 | 3,2 | 9 | 0.0036 | 0,0234 | 1.5817 |
| Cell Cycle Mitotic | 409 | 2,3 | 6 | 0.0253 | 0,0367 | 1.467 |
| Immune System | 942 | 5,3 | 13 | 0.0013 | 0,0164 | 1.38 |

FDR: False Discovery Rate

**Supplementary Table 7.** Results of pathway enrichment analyzing the EV-miRNAs under expressed in patients with worse outcome , together with their correlated miRNAs

| **Pathway** | **Total** | **Expected** | **Hits** | **p-value** | **FDR** | **RichScore** |
| --- | --- | --- | --- | --- | --- | --- |
| Negative regulation of the PI3K/AKT  network | 6 | 0,0747 | 2 | 0.0022 | 0.0047 | 33.3333 |
| Loss of Function of SMAD2/3 in  Cancer | 7 | 0,0871 | 2 | 0.0031 | 0.0057 | 28.5714 |
| Loss of Function of TGFBR1 in  Cancer | 7 | 0,0871 | 2 | 0.0031 | 0.0057 | 28.5714 |
| Post-transcriptional silencing by  small RNAs | 7 | 0,0871 | 2 | 0.0031 | 0.0057 | 28.5714 |
| Signaling by TGF-beta Receptor  Complex in Cancer | 8 | 0,0996 | 2 | 0.0041 | 0.0068 | 25 |
| G beta:gamma signalling through  PI3Kgamma | 8 | 0,0996 | 2 | 0.0041 | 0.0068 | 25 |
| CD28 dependent PI3K/Akt signaling | 15 | 0,187 | 3 | 0.0008 | 0.0019 | 20 |
| Constitutive Signaling by AKT1 E17K  in Cancer | 22 | 0,274 | 4 | 0.0001 | 0.0005 | 18.1818 |
| Downregulation of ERBB2:ERBB3  signaling | 11 | 0,137 | 2 | 0.0078 | 0.0112 | 18.1818 |
| AKT phosphorylates targets in the  cytosol | 11 | 0,137 | 2 | 0.0078 | 0.0112 | 18.1818 |
| CTLA4 inhibitory signaling | 11 | 0,137 | 2 | 0.0078 | 0.0112 | 18.1818 |
| G-protein beta:gamma signalling | 11 | 0,137 | 2 | 0.0078 | 0.0112 | 18.1818 |
| Pre-NOTCH Transcription and  Translation | 19 | 0,236 | 3 | 0.0016 | 0.0036 | 15.7895 |
| Activation of BAD and translocation  to mitochondria | 13 | 0,162 | 2 | 0.0109 | 0.0145 | 15.3846 |
| Oncogene Induced Senescence | 30 | 0,373 | 4 | 0.0005 | 0.0013 | 13.3333 |
| VEGFR2 mediated vascular  permeability | 23 | 0,286 | 3 | 0.0028 | 0.0056 | 13.0435 |
| Pre-NOTCH Expression and  Processing | 24 | 0,299 | 3 | 0.0031 | 0.0057 | 12.5 |
| ARMS-mediated activation | 16 | 0,199 | 2 | 0.0164 | 0.021 | 12.5 |
| Conversion from APC/C:Cdc20 to  APC/C:Cdh1 in late anaphase | 16 | 0,199 | 2 | 0.0164 | 0.021 | 12.5 |
| RHO GTPases Activate ROCKs | 16 | 0,199 | 2 | 0.0164 | 0.021 | 12.5 |
| KSRP destabilizes mRNA | 17 | 0,212 | 2 | 0.0184 | 0.0227 | 11.7647 |
| Inflammasomes | 17 | 0,212 | 2 | 0.0184 | 0.0227 | 11.7647 |
| Downregulation of TGF-beta  receptor signaling | 26 | 0,324 | 3 | 0.0039 | 0.0068 | 11.5385 |
| CD28 co-stimulation | 26 | 0,324 | 3 | 0.0039 | 0.0068 | 11.5385 |
| Integrin alphaIIb beta3 signaling | 27 | 0,336 | 3 | 0.0044 | 0.0072 | 11.1111 |
| Inhibition of the proteolytic activity of APC/C required for the onset of anaphase by mitotic spindle  checkpoint components | 18 | 0,224 | 2 | 0.0206 | 0.0242 | 11.1111 |
| Inactivation of APC/C via direct  inhibition of the APC/C complex | 18 | 0,224 | 2 | 0.0206 | 0.0242 | 11.1111 |

| SHC-related events triggered by  IGF1R | 18 | 0,224 | 2 | 0.0206 | 0.0242 | 11.1111 |
| --- | --- | --- | --- | --- | --- | --- |
| GPVI-mediated activation cascade | 28 | 0,348 | 3 | 0.0049 | 0.0077 | 10.7143 |
| SMAD2/SMAD3:SMAD4  heterotrimer regulates transcription | 28 | 0,348 | 3 | 0.0049 | 0.0077 | 10.7143 |
| Mitotic Spindle Checkpoint | 19 | 0,236 | 2 | 0.0228 | 0.0253 | 10.5263 |
| Prolonged ERK activation events | 20 | 0,249 | 2 | 0.0252 | 0.0265 | 10 |
| Metabolism of nitric oxide | 20 | 0,249 | 2 | 0.0252 | 0.0265 | 10 |
| eNOS activation and regulation | 20 | 0,249 | 2 | 0.0252 | 0.0265 | 10 |
| APC/C:Cdc20 mediated degradation  of Cyclin B | 21 | 0,261 | 2 | 0.0276 | 0.0288 | 9.5238 |
| TGF-beta receptor signaling  activates SMADs | 32 | 0,398 | 3 | 0.0071 | 0.0109 | 9.375 |
| Transcriptional activity of  SMAD2/SMAD3:SMAD4 heterotrimer | 43 | 0,535 | 4 | 0.0019 | 0.0042 | 9.3023 |
| Downregulation of SMAD2/3:SMAD4 transcriptional  activity | 22 | 0,274 | 2 | 0.0301 | 0.0304 | 9.0909 |
| Circadian Clock | 35 | 0,436 | 3 | 0.0091 | 0.0125 | 8.5714 |
| PI3K events in ERBB4 signaling | 94 | 1,17 | 8 | 1,92E-05 | 0.0002 | 8.5106 |
| PIP3 activates AKT signaling | 94 | 1,17 | 8 | 1,92E-05 | 0.0002 | 8.5106 |
| PI3K events in ERBB2 signaling | 94 | 1,17 | 8 | 1,92E-05 | 0.0002 | 8.5106 |
| PI-3K cascade:FGFR1 | 94 | 1,17 | 8 | 1,92E-05 | 0.0002 | 8.5106 |
| PI-3K cascade:FGFR2 | 94 | 1,17 | 8 | 1,92E-05 | 0.0002 | 8.5106 |
| PI-3K cascade:FGFR3 | 94 | 1,17 | 8 | 1,92E-05 | 0.0002 | 8.5106 |
| PI-3K cascade:FGFR4 | 94 | 1,17 | 8 | 1,92E-05 | 0.0002 | 8.5106 |
| Signaling by TGF-beta Receptor  Complex | 72 | 0,896 | 6 | 0,0003 | 0.0008 | 8.3333 |
| PI3K/AKT activation | 97 | 1,21 | 8 | 2,42E-05 | 0.0003 | 8.2474 |
| GAB1 signalosome | 98 | 1,22 | 8 | 2,61E-05 | 0.0003 | 8.1633 |
| Platelet Aggregation (Plug  Formation) | 37 | 0,461 | 3 | 0,0107 | 0.0145 | 8.1081 |
| Role of LAT2/NTAL/LAB on calcium  mobilization | 103 | 1,28 | 8 | 3,75E-05 | 0.0003 | 7.767 |
| Unfolded Protein Response (UPR) | 73 | 0,909 | 5 | 0,0021 | 0.0045 | 6.8493 |
| Signaling by SCF-KIT | 133 | 1,66 | 9 | 3,60E-05 | 0.0003 | 6.7669 |
| Downstream signal transduction | 151 | 1,88 | 10 | 1,56E-05 | 0.0002 | 6.6225 |
| Downstream signaling of activated  FGFR1 | 139 | 1,73 | 9 | 5,11E-05 | 0.0003 | 6.4748 |
| Downstream signaling of activated  FGFR2 | 139 | 1,73 | 9 | 5,11E-05 | 0.0003 | 6.4748 |
| Downstream signaling of activated  FGFR3 | 139 | 1,73 | 9 | 5,11E-05 | 0.0003 | 6.4748 |
| Downstream signaling of activated  FGFR4 | 139 | 1,73 | 9 | 5,11E-05 | 0.0003 | 6.4748 |
| Signaling by ERBB4 | 143 | 1,78 | 9 | 6,38E-05 | 0.0003 | 6.2937 |
| PI3K/AKT Signaling in Cancer | 81 | 1,01 | 5 | 0,0032 | 0.0058 | 6.1728 |
| Signaling by FGFR | 151 | 1,88 | 9 | 9,74E-05 | 0.0004 | 5.9603 |

| Signaling by FGFR1 | 151 | 1,88 | 9 | 9,74E-05 | 0.0004 | 5.9603 |
| --- | --- | --- | --- | --- | --- | --- |
| Signaling by FGFR2 | 151 | 1,88 | 9 | 9,74E-05 | 0.0004 | 5.9603 |
| Signaling by FGFR3 | 151 | 1,88 | 9 | 9,74E-05 | 0.0004 | 5.9603 |
| Signaling by FGFR4 | 151 | 1,88 | 9 | 9,74E-05 | 0.0004 | 5.9603 |
| Signaling by ERBB2 | 152 | 1,89 | 9 | 0,0001 | 0.0004 | 5.9211 |
| Fc epsilon receptor (FCERI) signaling | 169 | 2,1 | 10 | 4,14E-05 | 0.0003 | 5.9172 |
| Nuclear Receptor transcription  pathway | 51 | 0,635 | 3 | 0,0252 | 0.0265 | 5.8824 |
| DAP12 signaling | 154 | 1,92 | 9 | 0,0001 | 0.0004 | 5.8442 |
| Signaling by PDGF | 177 | 2,2 | 10 | 6,15E-05 | 0.0003 | 5.6497 |
| Cellular Senescence | 143 | 1,78 | 8 | 0,0004 | 0.0011 | 5.5944 |
| Costimulation by the CD28 family | 54 | 0,672 | 3 | 0,0293 | 0.0299 | 5.5556 |
| Downstream signaling events of B  Cell Receptor (BCR) | 164 | 2,04 | 9 | 0,0002 | 0.0006 | 5.4878 |
| Transcriptional Regulation by TP53 | 55 | 0,685 | 3 | 0,0307 | 0.0307 | 5.4545 |
| VEGFA-VEGFR2 Pathway | 92 | 1,15 | 5 | 0,0056 | 0.0087 | 5.4348 |
| Signaling by EGFR | 168 | 2,09 | 9 | 0,0002 | 0.0007 | 5.3571 |
| NGF signalling via TRKA from the  plasma membrane | 189 | 2,35 | 10 | 0,0001 | 0.0004 | 5.291 |
| Generic Transcription Pathway | 189 | 2,35 | 10 | 0,0001 | 0.0004 | 5.291 |
| DAP12 interactions | 171 | 2,13 | 9 | 0,0003 | 0.0008 | 5.2632 |
| Signaling by VEGF | 100 | 1,24 | 5 | 0,0079 | 0.0112 | 5 |
| RHO GTPases Activate Formins | 102 | 1,27 | 5 | 0.0086 | 0.0119 | 4.902 |
| IRS-related events triggered by  IGF1R | 84 | 1,05 | 4 | 0.0203 | 0.0242 | 4.7619 |
| Signaling by the B Cell Receptor  (BCR) | 190 | 2,36 | 9 | 0.0005 | 0.0014 | 4.7368 |
| Signaling by NOTCH | 86 | 1,07 | 4 | 0.0219 | 0.0253 | 4.6512 |
| Signaling by Type 1 Insulin-like  Growth Factor 1 Receptor (IGF1R) | 87 | 1,08 | 4 | 0.0228 | 0.0253 | 4.5977 |
| IGF1R signaling cascade | 87 | 1,08 | 4 | 0.0228 | 0.0253 | 4.5977 |
| Insulin receptor signalling cascade | 87 | 1,08 | 4 | 0.0228 | 0.0253 | 4.5977 |
| Oxidative Stress Induced Senescence | 88 | 1,1 | 4 | 0.0236 | 0.0259 | 4.5455 |
| Diseases of signal transduction | 235 | 2,92 | 10 | 0.0006 | 0.0016 | 4.2553 |
| Cell Cycle Checkpoints | 123 | 1,53 | 5 | 0.0182 | 0.0227 | 4.065 |
| Signalling by NGF | 273 | 3,4 | 11 | 0.0005 | 0.0014 | 4.0293 |
| Cellular responses to stress | 256 | 3,19 | 10 | 0.0012 | 0.0029 | 3.9063 |
| RHO GTPase Effectors | 234 | 2,91 | 9 | 0.0024 | 0.0049 | 3.8462 |
| Adaptive Immune System | 430 | 5,35 | 14 | 0.0008 | 0.0019 | 3.2558 |
| Innate Immune System | 569 | 7,08 | 16 | 0.0015 | 0.0035 | 2.812 |
| Signaling by Rho GTPases | 349 | 4,34 | 9 | 0.0287 | 0.0296 | 2.5788 |
| Immune System | 942 | 11,7 | 24 | 0.0003 | 0.001 | 2.5478 |
| Gene Expression | 851 | 10,6 | 19 | 0.0073 | 0.0111 | 2.2327 |

FDR: False Discovery Rate
